# Image facilitated assessment of intra-spike variation in grain size in wheat under high temperature and drought stress

**DOI:** 10.1038/s41598-023-44503-x

**Published:** 2023-11-13

**Authors:** Vidisha Thakur, Jagadish Rane, Girish Chandra Pandey, Satish Yadav

**Affiliations:** 1 Department of Bioscience & Biotechnology, Banasthali Vidyapith, Banasthali, Rajasthan 304 022 India; 2 ICAR-Central Institute for Arid Horticulture, Bikaner, Rajasthan 334006 India; 3 ICAR-Directorate of Onion and Garlic Research, Rajgurunagar, Pune, 410 505 India

**Keywords:** Biotechnology, Plant sciences

## Abstract

In wheat (*Triticum aestivum* L.), the grain size varies according to position within the spike. Exposure to drought and high temperature stress during grain development in wheat reduces grain size, and this reduction also varies across the length of the spike. We developed the phenomics approach involving image-based tools to assess the intra-spike variation in grain size. The grains were arranged corresponding to the spikelet position and the camera of smart phone was used to acquire 333 images. The open-source software ImageJ was used to analyze features of each grain and the image-derived parameters were used to calculate intra-spike variation as standard deviation (ISVAD). The effect of genotype and environment were highly significant on the ISVAD of grain area. Sunstar and Raj 4079 contrasted in the ISVAD of grain area under late sown environment, and RNA sequencing of the spike was done at 25 days after anthesis. The genes for carbohydrate transport and stress response were upregulated in Sunstar as compared to Raj 4079, suggesting that these play a role in intra-spike assimilate distribution. The phenomics method developed may be useful for grain phenotyping and identifying germplasm with low intra-spike variation in grain size for their further validation as parental material in breeding.

## Introduction

The number of developed grains and the grain weight are the critical components of grain yield which have been extensively used for the improvement of productivity in wheat (*Triticum aestivum* L.). Variation exists in these two traits within the spike. The proximal florets and middle spikelets have larger grains than the distal florets and top spikelets^1–3^. The distal primordia are prominently delayed with respect to the rate of initiation exhibited by proximal primordia, however, the maturation of all grains is synchronous which may cause less assimilate supply to distal grains^4,5^. The uneven intra-spike assimilate distribution is demonstrated from the reports that approximately 44% of the total number of spikelets located in the lower half of the spike account for 64% of the grain yield^6^. The source or photosynthetic potential is underutilized without the efficient partitioning of assimilates to sink (grains)^7–9^, therefore, increasing the sink strength is critical for further improving wheat productivity^10^. Breeding for the sink strength (grain number or potential weight) has continuously boosted wheat yield previously^11^.

A uniform intra-spike grain filling is preferred to obtain higher yield and better wheat quality. Grain yield is greatly decreased because smaller grains are readily lost during the winnowing process before milling^12,13^. Grain size variation also increases the variability of seedling emergence, early groundcover, and initial biomass, indirectly reducing crop management efficiency and production^14,15^. Heterogeneity of grain mass deteriorates flour quality and end-use suitability^16^.

Individual grain size variance at intra-genotype level is influenced by specific chromosomal regions and the assimilate translocation to grains^17^. At a temperature above 35 °C, the grain size is reduced due to less starch content in grains^18^, and the reduction in single grain weight can be as high as 20.61% if the high temperature prevails from the start of grain filling until ripening^19^. Reduced photosynthesis and increased leaf senescence under post-anthesis high temperatures restrict the availability of starch for deposition and usually cause top and bottom spikelets and distal florets to either abort or produce small grains^20–22^. An earlier study reported that post anthesis temperature regime of 30/25 °C decreases grain size in all floret positions within the spike^23^. Later it was found that grains located on different parts of the spike and spikelets have distinct responses to post-anthesis high temperature^24^. Several studies on diverse germplasm observed genetic variability in grain size stability in response to heat stress^25,26^. In addition, soil moisture deficit during grain filling increases the small-sized grains; however, breeding can reduce this risk^27^.

The limitations of traditional genetic improvement methods primarily focused on enhancing grain yield have prompted the adoption of genomics-driven tools, including genomic selection. These tools hold the potential to revolutionize plant breeding processes by increasing precision and efficiency^28^. Integrating phenomics protocols is crucial for maximizing the benefits of genomic science in advancing crop improvement. Furthermore, the often overlooked intraspike assimilate distribution in wheat's grain yield dynamics emphasizes the significance of sophisticated imaging techniques such as X-ray CT (Computed Tomography) and NMR (Nuclear Magnetic Resonance)^29–32^. However, due to their high cost, these tools are not accessible to many crop scientists. In response to this limitation, efforts have been made to develop an affordable phenotyping approach for intraspike assimilate distribution. The phenotyping approach aims to provide a cost-effective alternative for studying this intricate plant trait, ensuring that a wider range of researchers can contribute to advancing crop science. Images of grains within the spike were captured using smartphone camera for sixteen diverse wheat genotypes grown under non-stressed, terminal drought and terminal high temperature environments and analyzed using an open source software ImageJ. Our approach accurately extracted many agronomically relevant grain size and shape parameters of each grain within the spike that was used to calculate the intra-spike variation as standard deviation (ISVAD). This method was used to quantify the genetic variation and effect of genotype-environment interaction on the intra-spike variation in grain size. The RNA sequencing of the spike of contrasting genotypes was conducted to identify the genes responsible for low intra-spike variation in grain size.

## Results

### Validation of ImageJ results

**Figure 1:**
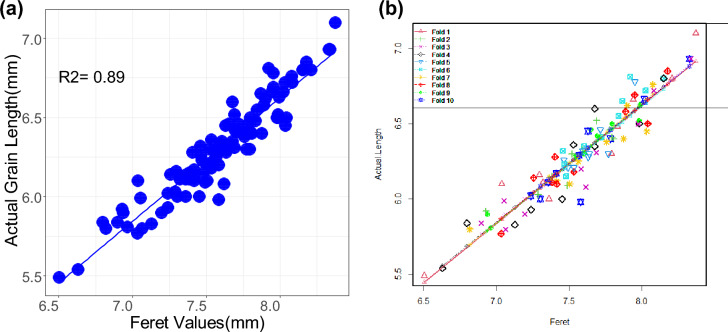
(**a**) Prediction of actual grain length measured by vernier calliper (mm) and those predicted by *Feret* values obtained from ImageJ analysis (**b**) and tenfold cross validation by DAAG software. The symbols of different shapes and colors show cross-validation predicted values.

**Figure 2:**
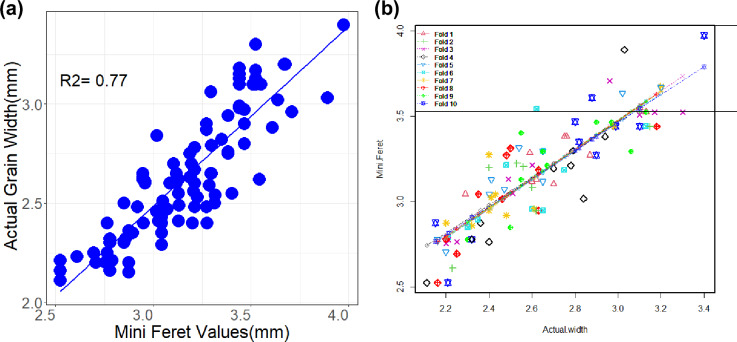
(**a**) Prediction of actual grain width measured by vernier calliper (mm) and those predicted by *MinFeret* values obtained from ImageJ analysis (**b**) and tenfold cross validation by DAAG software. The symbols of different shapes and colors show cross-validation predicted values.

The known length in millimeters of the line drawn against the scale in the first image is entered in the *set scale* tool which converts the pixels into mm and this calibration applies to all the images processed in the session. The distance between the grains and the camera was fixed at 31 cm for all the images. The manually counted actual number of grains spike^−1^ was same as the number of grains values given by ImageJ. The ImageJ result parameters, *Feret* and *MinFeret* of each grain could explain the actual grain length and width in the selected genotypes. The *Feret* and *MinFeret* values obtained from ImageJ correlated to the actual length and width of 100 grains as measured by the vernier caliper (R^2^ = 0.892 and R^2^ = 0.77) (Figs. 1a, 2a). The correlation of actual grain length with *Feret* values and actual grain width with *MinFeret* values was the same in a repeat of the experiment. Therefore, we confirmed the consistency over grain size estimation by ImageJ. The predicted values were tenfold cross-validated using the cv.lm function of “DAAG” package of R^33^ (Figs. 1b, 2b). The function cv.lm carries out a k-fold cross-validation for a linear model (i.e. an 'lm' model). For each fold, an 'lm' model is fit to all observations that are not in the fold (the 'training set') and prediction errors are calculated for the observations in the fold (the 'test set'). The prediction errors are the absolute error and its square. The average prediction errors over the observations in the fold are calculated, and the square root of the average of the squared errors is taken.

### Intra-spike variation in grain size

The genotypes were sown for the non-stress or control (C) and terminal drought (D) during the optimal sowing period, commonly known as timely sown in India (usually referred to as free from heat stress)^34^. The sowing between the mid of December to last week is referred to as late sown (L) in the India when the crop is naturally exposed to terminal high-temperature stress^34^. Heading and anthesis occurred in the third week of February under C and D environments, and in the first week of March under L environment. Physiological maturity occurred at the end of March under all environments. The mean daily maximum temperature from heading to maturity under timely sown conditions (C, D) and late sown condition (L) was 30.04 °C and 32.21 °C in 2018–2019; 30.72 °C and 31.79 °C in 2019–2020 and, 34.32 °C and 36.18 °C in 2020–2021 (Table 3). This was conducive to create measurable variations in the grain size for the optimization of methods. The grain yield plot^−1^ was correlated to grain yield spike^−1^ (R^2^ = 0.456) (Fig. S1). The measurements of the spike yield components (grains spike^−1^, grain yield spike^−1^, spikelets spike^−1^) and spike length were done using three replicates of main stem spikes only; however the grain yield plot^−1^ includes the spikes of main stems and tillers.

**Figure 3:**
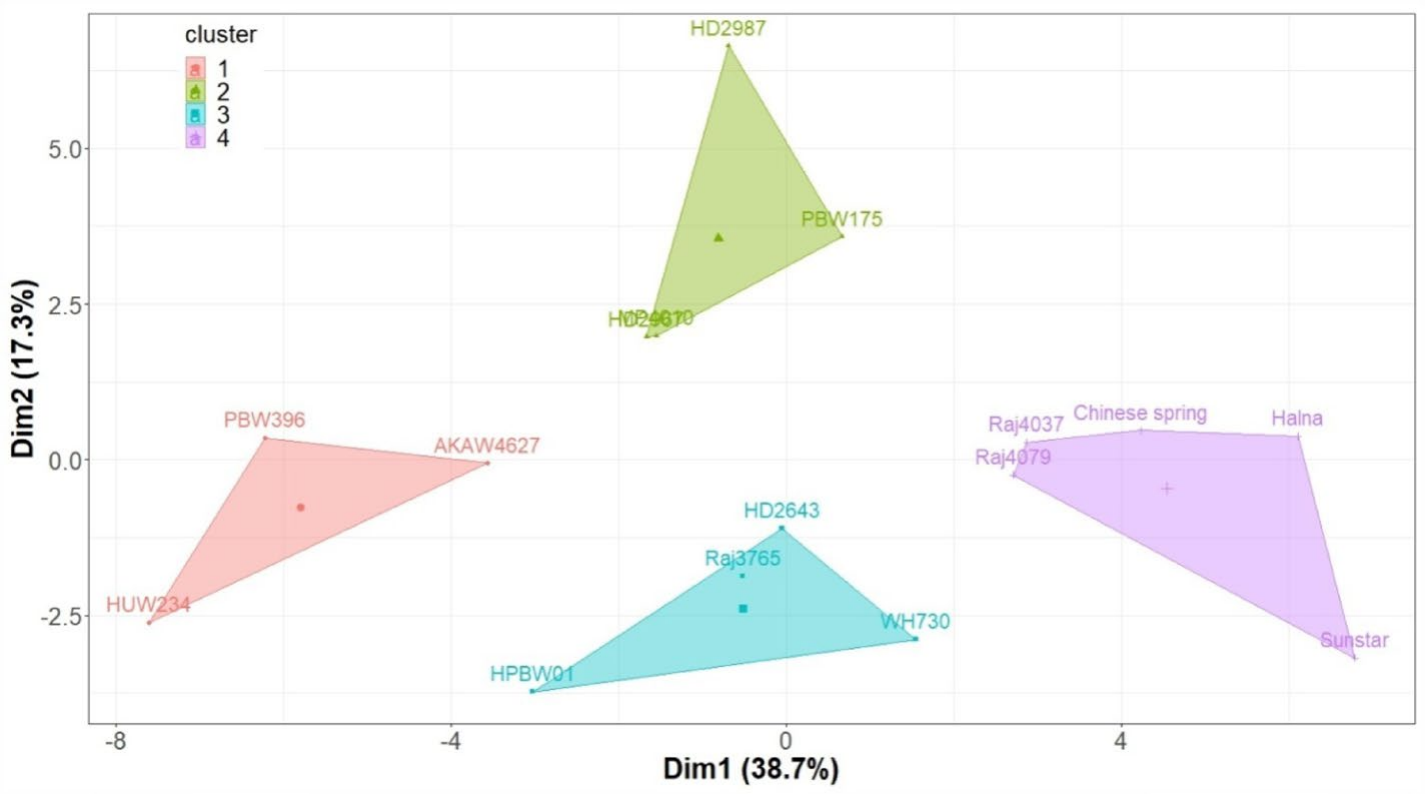
K means-Cluster analysis of spike yield components (spikelets spike^−1^, grains spike^−1^ and grain yield spike^−1^) and spike length under C, D and L during 2018–2019 and 2019–2020.

The grain area ranged from 3.04 to 30.15 mm^2^ across all the genotypes and environments. The environments C, D and L had a significant effect on the ISVAD of grain width (p ≤ 0.001) and area (p ≤ 0) in 2018–2019 and 2019–2020 (Table S1). The ISVAD of grain area and grain width were highly influenced by genotype and genotype-environment interaction during 2018–2019 and 2019–2020 (Table S1).The mean ISVAD of grain width across the genotypes was higher under C (0.366^a^) as compared to D (0.354^b^) and L (0.34^c^) in 2018–2019 and 2019–2020 (Table 1). The mean ISVAD of grain area also decreased under D (2.9^b^) and L (2.786^c^) as compared to C (3.21^a^). The grains spike^−1^, grain yield spike^−1^, single grain weight (SGW), spikelets spike^−1^ and spike length decreased significantly under D and L in comparison to C (Table 1). The mean grain length/width ratio increased under D (2.26^b^) and L (2.215^b^) as compared to C (2.137^a^) and the mean SGW (g) decreased under D (0.041^b^) and L (0.039^b^) compared to C (0.045^a^) (Table 1). The K mean cluster plot for 56% variation of spike length, spikelets spike^−1^, grains spike^−1^ and yield spike^−1^ under C, D and L environments of 2018–2019 and 2019–2020 gives four clusters of genotypes (Fig. 3). Within the cluster 4, Sunstar has significantly lower mean ISVAD of grain area as compared to Raj 4079 and Raj 4037 across C, D and L environments (Tables S2–S4). Under C, the mean SGW (g) of 2018–2019 and 2019–2020 of Sunstar (0.038^d^) is lower as compared to Raj 4079 (0.051^ab^), however under D the SGW (g) of Sunstar (0.037^ab^) is higher than Raj 4079 (0.025^c^). Under L environment, the SGW (g) of Sunstar (0.025^ef^) and Raj 4079 (0.035^de^) are comparable and the ISVAD of grain area of Sunstar (1.778^e^) is notably lower than Raj 4079 (2.696^bcd^).

**Table 1:** The effect of drought and high temperature prevailed during control, terminal drought and late sown conditions on the mean values of grain parameters across 16 genotypes evaluated during 2018–2019 and 2019–2020.

Parameter	Control	Terminal drought	Late sown
ISVAD of grain width	0.366^a^	0.354^b^	0.34^c^
ISVAD of grain length	0.617^a^	0.606^a^	0.59^b^
ISVAD of grain area	3.21^a^	2.9^b^	2.786^c^
ISVAD of grain perimeter	1.688^a^	1.659^a^	1.613^a^
Grain length**/**width ratio	2.137^b^	2.26^a^	2.215^a^
Grains spike^−1^	62^a^	54^b^	54^b^
Yield spike^−1^ (g)	2.71^a^	2.14^b^	2.1^b^
SGW (g)	0.045^a^	0.041^b^	0.039^b^
Spikelets spike^−1^	20^a^	18^b^	18^b^
Spike length (cm)	11.5^a^	10.6^b^	10.1^c^

The letters^(a–c)^ suffixed with the mean values of 96 observations (16 genotypes × 3 replications × 2 years) were derived from Duncan’s multiple range test (p < 0.05) and the means with different letters within each row are significantly different.

**Figure 4:**
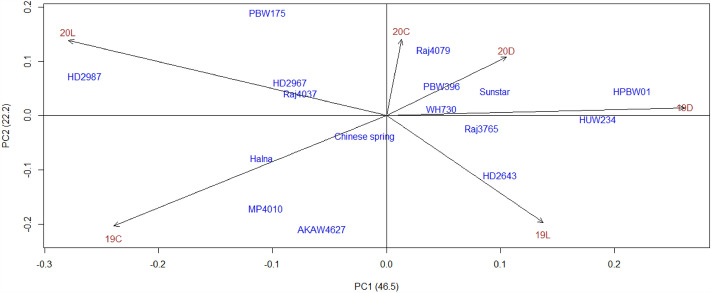
Biplot of genotype and environment for intra-spike variation as standard deviation (ISVAD) of grain area across the environments in 2018–2019 and 2019–2020. Where, 19- 2018–2019; 20- 2019–2020; C-Control, D-Drought and L-Late sown.

**Figure 5:**
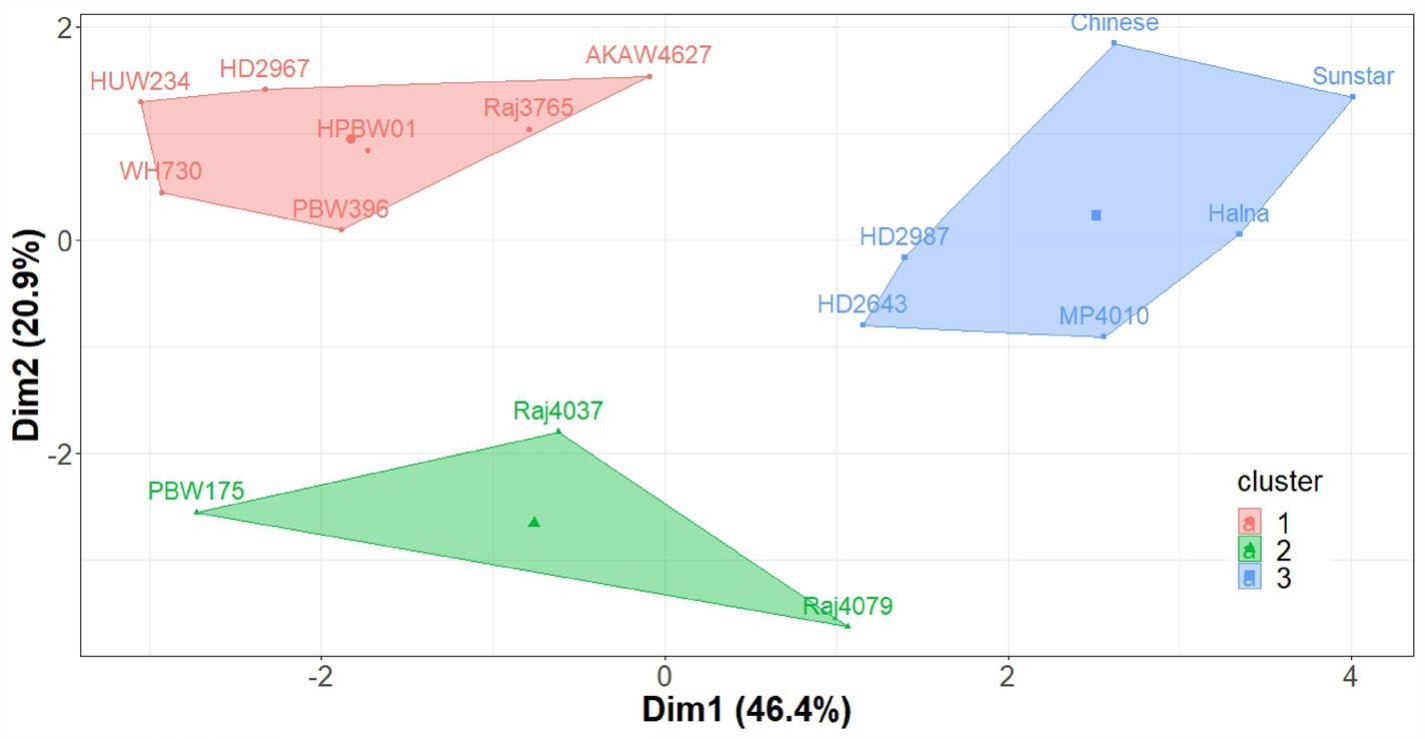
K means-Cluster analysis of rachis area and perimeter of sixteen genotypes across control, drought and late sown environments of 2018–2019 and 2019–2020.

Biplot accounting for 68.7% variation reveals that ISVAD of grain area of HD 2987, HPBW 01, PBW 175, AKAW 4627, HUW 234 and MP 4010 is highly influenced by the environments (Fig. 4). Among the 16, HD 2987 had the highest ISVAD of grain area under C (4.011) and the grain yield spike^−1^ of HD 2987 decreased significantly under D and L (Tables S2–S4). The grains spike^−1^ of HD 2643 and HD 2967 decreased markedly under D and L as compared to the C. The genotypes Chinese spring, PBW 396 and WH 730 have most stable ISVAD of grain area; however the three genotypes are different in spike yield components (Figs. 3, 4). PBW 396 is distinct from Chinese spring and WH 730 in the distance heat map of the ISVAD of grain width, length, area and perimeter across the environments in 2018–2019 and 2019–2020 (Fig. S2), as PBW 396 has a high ISVAD of grain area across these 6 environments (Tables S2–S4). The compact spike shaped genotypes Chinese spring, AKAW 4627, Sunstar and HUW 234 are similar in the ISVAD of grain width, length, area and perimeter as represented by the distance heat map. The K means-cluster plot of rachis area and rachis perimeter under C, D and L environments, accounting for 67.3% variation formed three different clusters of genotypes, where Raj 4079, PBW 175 and Raj 4037 are in cluster 2, whereas Sunstar, Chinese Spring, Halna, HD 2987, HD 2643 and MP 4010 are in cluster 3 (Fig. 5).

**Figure 6:**
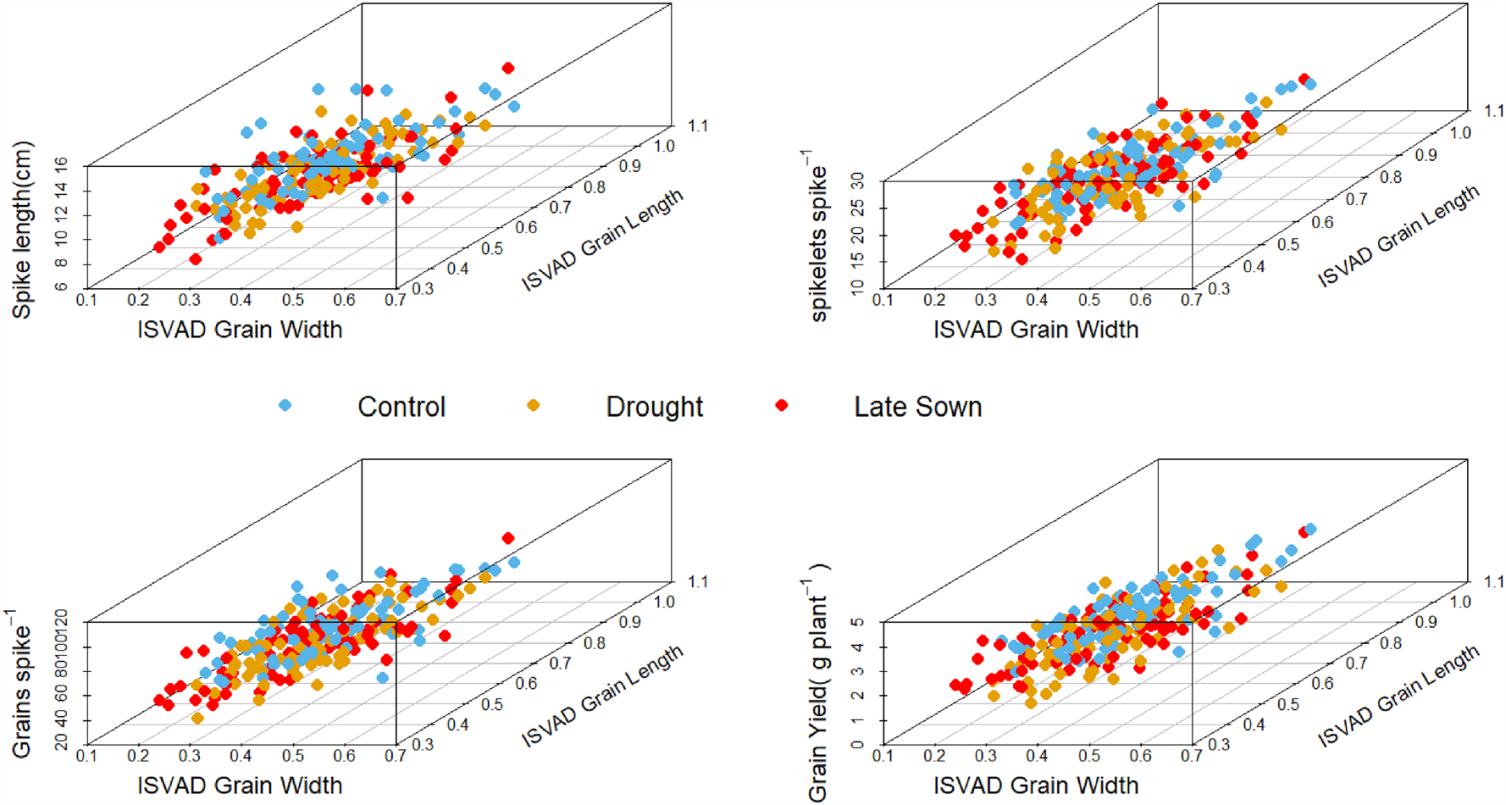
The effect of (**a**) spike length (**b**) spikelets spike^−1^ (**c**) grains spike^−1^ and (**d**) grain yield spike^−1^ on the association between ISVAD of grain length and ISVAD of grain width in control, drought and late sown environments of 2018–2019 and 2019–2020.

The top region of spike has relatively higher ISVAD of grain width and ISVAD of grain area than middle and bottom regions under C (Fig. S3a,b) and also across the C, D and L environments (Fig. S4a,b) of 2018–2019 and 2019–2020. The effect of environment was highly significant on the ISVAD of grain area within top (p < 0.001) and bottom (p < 0.001) regions of spike whereas it was less significant on the middle (p < 0.05) region of spike (Table S5). However, the genotype-environment interaction influenced only top and bottom ISVAD of grain area. The ISVAD of grain width was influenced by environment only in the middle (p < 0.05) and bottom (p < 0.01) regions of the spike. The genotype-environment interaction notably affects only the bottom region ISVAD of grain width (Table S6). The ISVAD of grain length was weakly associated with ISVAD of grain width; however, D and L impaired this relationship (Fig. 6). The ISVAD of grain width and length was not influenced by the spike length, spikelets spike^−1^, grains spike^−1^ and grain yield spike^−1^. The Fig. 6 also depicts that the environment has no effect on the association between ISVAD of grain length and spike yield components.

**Figure 7:**
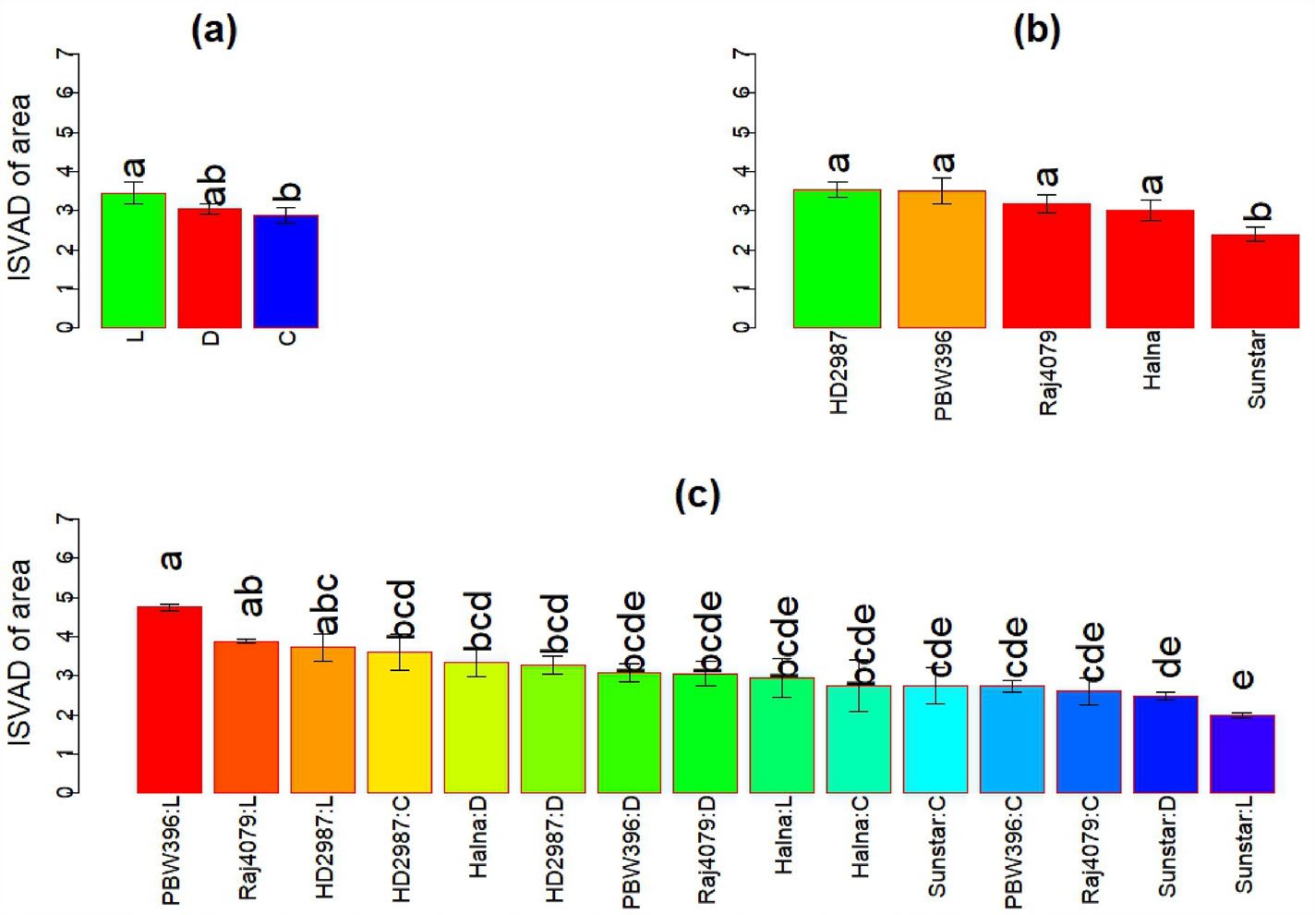
Variation in intra-spike variation as standard deviation (ISVAD) of grain area. (**a**) The effect of late sown (L), terminal drought (D) and Control (C) environments on ISVAD of grain area in 2020–2021. Each bar in the environment effect represents 15 observations (3 replications and 5 genotypes). (**b**) The genotype effect across the environments on ISVAD of grain area. Each bar in the genotype effect represents 9 observations (mean values of three replications and three environments). (**c**) Genotype environment interaction effect on ISVAD of grain area. Each bar in the genotype environment interaction effect represents mean values of three replications. Letters represent the significance of differences among mean values as computed by the Duncan multiple range test at p ≤ 0.05. Genotypes with common letters are not significantly different.

The experiments were repeated in 2020–2021 for five genotypes Sunstar, Halna, Raj 4079, HD 2987 and PBW 396. The crop season 2020–2021 had highest mean maximum daily temperature of 41.6 °C (Table 3). The ISVAD of grain area in 2020–2021 increased significantly under L (Fig. 7) although grains spike^−1^ decreased as compared to C (Fig. S5). The grain yield spike^−1^ decreased under D and L (Fig. S6). The ISVAD of grain area did not change significantly in D but the grains spike^−1^ was reduced. The mean ISVAD of grain area was significantly lower in Sunstar (2.401^b^), as compared to Raj 4079 (3.185^a^) (Fig. 7). The effect of environment was significant only on ISVAD of grain area of selected five genotypes in three crop seasons (Table S7).

In summary, the effects of genotype and environment were significant on the ISVAD of grain area across the three crop seasons (Tables S1, S7). The grains spike^−1^, grain yield spike^−1^, SGW, spikelets spike^−1^ and spike length decreased significantly under D and L in comparison to C. In the first two years, ISVAD of grain area also decreased under D and L (Table 1). The K-means cluster plot of spike length and spike yield components formed 4 clusters. Within cluster 4, Sunstar and Raj 4079 have significantly different ISVAD of grain area. The ISVAD of grain area was higher in the top region of the spike as compared to middle and bottom regions of spike across the environments (Fig. S4b). The effect of environment and genotype-environment interaction was significant on top and bottom regions of spike (Table S5). In 2020–2021, ISVAD of grain area increased, and grains spike^−1^ and grain yield spike^−1^ decreased (Figs. 7, S5, S6).

### Sequence assembly based on cDNA libraries sequencing data

The RNA sequencing of three biological replicates of Sunstar under L (SL) and Raj 4079 under L (RL) generated 160 million and 170 million reads (Table S8). The sequencing raw data have been submitted to NCBI and the accession number is PRJNA918542. GC content and Q30 is 52.20 and 87.50% for SL, and 53.28% and 93.09% for RL respectively. Q30 refer to a 0.1% chance of error and 99.9% confidence. These results indicate that all the data were qualified for downstream analysis.

### Differential expression of genes (DEGs)

**Figure 8:**
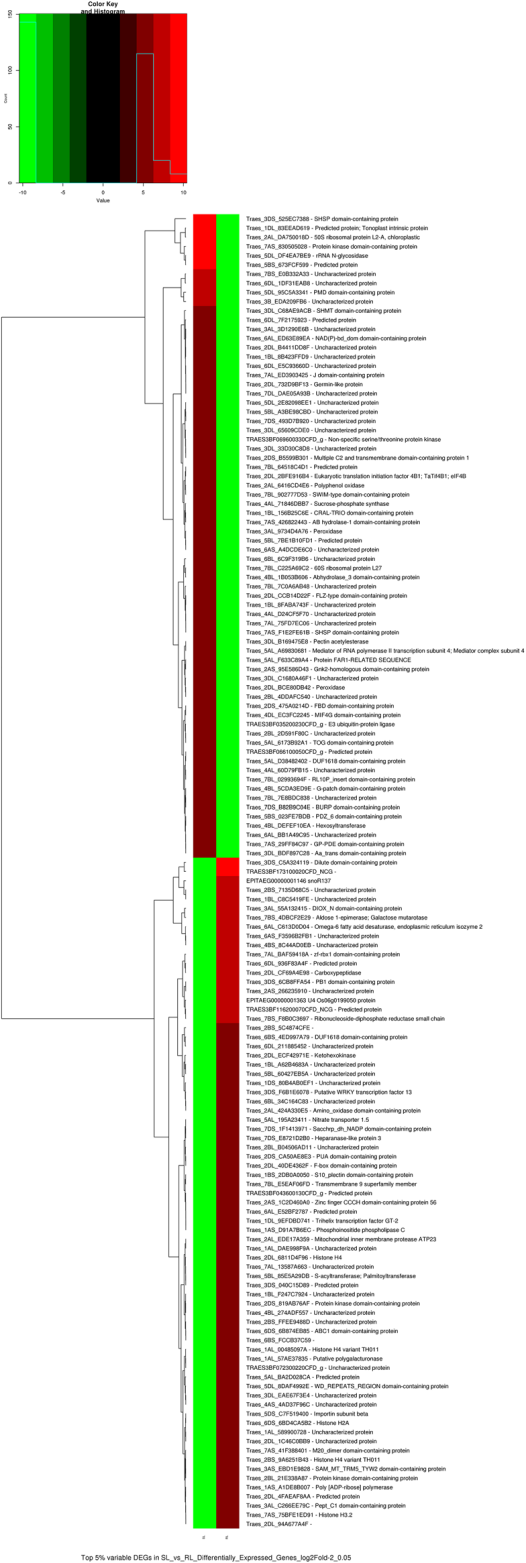
Heat map of top 5% differentially expressed genes log2 fold 2 at p < 0.05 between Sunsar and Raj 4079 under late sown (SL and RL). Heat map was generated with the R (Version 3.4.4) function *heatmap.2()* of the package gplots https://CRAN.R-project.org/package=gplots^35^.

A total of 2861 DEGs were identified out of which 1582 DEGs were downregulated in RL and 1279 were upregulated in RL as compared to SL at p < 0.05. The top 5% DEGs are shown by heat map (Fig. 8).

### Functional analysis of DEGs

Gene Ontology (GO) analysis was used to analyze functions of DEGs. These DEGs are classified into 54 functional groups in three main categories (cellular component, molecular function and biological process) (Figs. S7–S9). GO annotation results showed that the terms of cell anatomical structure and organelle were dominant in the cellular component, those of binding and catalytic activity were primary in molecular function, and those of metabolic process, and cellular process were foremost in biological process. These GO terms demonstrated that genes related to wheat late grain development encoded diverse regulators and protein. Among the 92 DEGs involved in carbohydrate transport and metabolism, 34 DEGs were involved in sucrose transport and sucrose metabolism. 97 transmembrane transporters genes were differentially expressed between Sunstar and Raj 4079 under L environment. The Ubiquitin-proteasome system plays an important role in grain development during later stage as reflected by 43 DEGs related to ubiquitin-mediated proteolysis and proteasome pathway. The expression of 18 DEGs of signal transduction was significantly lower in Raj 4079. A total of 25 genes were downregulated in Raj 4079 in response to heat stress, water stress and oxidative stress. The genes involved in transmembrane transporter activity were downregulated under high temperature stress in Raj 4079 such as 3 SWEET bidirectional sugar transporter genes. Other transporter genes downregulated were peptide transporter PTR3-A, 10 ABC transporter C family member, 2 low affinity nitrate transporter NPF7.1a, 3 cadmium/zinc-transporting ATPase and 7 oligopeptide transporter. The sucrose phosphate synthase involved in sucrose synthesis and metabolism during grain development was also downregulated in Raj 4079 under high temperature stress. The stress responsive gene transcripts, temperature stress induced lipocalin, and stress induced transcription factor SNAC1 expression were downregulated in Raj 4079 as compared to Sunstar.

## Discussion

### Phenomics for wheat grain size

The limited progress achieved through traditional genetic improvement methods, primarily centered around enhancing grain yield, has prompted the utilization of tools stemming from advancements in genomics. These tools focus on dissecting individual traits that are associated with specific genes. Genomic selection, among these approaches, presents an alternative means of predicting breeding values in plants by leveraging genome-wide markers. This strategy holds the promise of expediting genetic advancement through heightened selection precision, accuracy, and a reduction in the time required for breeding cycles^28^. Nonetheless, the successful implementation of genomic selection necessitates the availability of both phenotypic and genomic data for a designated set of genotypes within a given crop. Thus, phenomics protocols emerge as pivotal components in harnessing the benefits of genomic science to drive further progress in crop improvement. While considerable attention has been directed towards understanding wheat grain yield constituents such as grain number and weight, certain critical factors, notably the distribution of assimilates within the spike (intraspike), have not received thorough investigation. This facet has the potential to significantly influence grain yield outcomes particularly under abiotic stress environments. Measuring intraspike assimilate distribution requires a meticulous characterization of each individual grain within the spike. However, this task becomes notably more intricate when dealing with a substantial number of genotypes that need screening. To address this complexity, sophisticated and costly tools have been demonstrated. These tools, including X-ray CT or NMR, offer advanced imaging capabilities, enabling the non-destructive examination of plant structures and traits such as features of grains^29,31,32^. However, these tools are not affordable for many of the crop improvement scientists. Hence an attempt was made to employ affordable phenotyping approach for assessing genetic variability in traits such as ISVAD. Our method uses simple equipment to collect images and processing of images is quick with an open source software ImageJ. It is fully-automated as the thresholding and make binary tool ensure the accurate detection of grains against the background. ImageJ provides a large number of complex traits, while sequentially numbering each grain and thereby retaining positional information of each grain in spike and spikelet. In addition to the grain size parameters such as feret’s diameter, area and perimeter, it also measures the shape descriptors such as circularity, aspect ratio and roundness (https://imagej.nih.gov/ij/docs/guide/146-30.html). Haghshenas et al. (2022) used ImageJ to explore the image-based mean grain weight estimations in wheat by developing simple predictive linear models^36^. ImageJ has been previously used to measure the grain size and shape for QTL analysis in wheat^37^ and Arabidopsis^38^. It has also been used for measurement of grain traits of rice^39^ and different food grains^40^. The wheat grain characteristics have also been explored using other image-based phenotyping platforms such as WinSEEDLE (Regent Instruments, Quebec City, QC, Canada)^41–46^, SeedCount (Next Instruments, Condell Park, Australia)^47–53^ and Marvin seed analyzer^54–56^ however, these are associated with expensive specialized hardware. SmartGrain and GrainScan are similar free softwares that require low-cost equipment and are more commonly used for grain phenotyping^57,58^, to explore genetic underpinnings of grain size^59–65^, to help breeding and introgression^66–68^, to explore drought response^69^ and evaluate genebank accessions^70^. In this study, the analysis of variance (ANOVA) of 16 genotypes in the first two crop seasons and combined ANOVA of three crop seasons for five genotypes confirmed the significant effect of genotype and environment on ISVAD of grain area. Therefore, the grain area was the most reliable parameter for the analysis of intra-spike variation in grain size. We show that ImageJ assessment of quantitative grain characteristics can be an accurate and affordable method for the estimation of ISVAD of grain size for large sample sizes. In summary, the limitations encountered in traditional genetic improvement pathways, which predominantly target grain yield enhancement, have spurred the adoption of genomics-driven tools. These tools, exemplified by genomic selection, have the potential to revolutionize breeding processes through enhanced precision and efficiency. The integration of phenomics protocols is pivotal in maximizing the benefits of genomic science for the advancement of crop improvement. Moreover, the overlooked aspect of intraspike assimilate distribution in wheat's grain yield dynamics underscores the importance of sophisticated imaging techniques like X-ray CT and NMR, which facilitate the investigation of intricate plant traits. Since these are expensive and not affordable for many of the crop scientists, an attempt was made to develop an affordable phenotyping approach for ISVAD.

### ISVAD as trait for assessment of sensitivity to high temperature and soil moisture stress

Genetic variation exists in ISVAD of grain width, length, area and perimeter within the spike. The ISVAD of grain area was highest in the top region of spike (Fig S3b, S4b), indicating that the assimilate distribution was uneven in top region. This is in agreement with an earlier report that increased temperature reduces grain volume at the top of the spike when water is non-limiting; however, a significant reduction in grain number occurs in the middle of the spike, with the top and bottom regions being less affected^71^. Under combined heat and drought stress, and drought stress alone, the grain weight reduces significantly at the top and bottom of the wheat spike^72^. The effect of genotype-environment interaction was significant on ISVAD of grain area of only the spike's top and bottom regions (Table S5), indicating that genotypes have distinct responses to grain size reduction in these regions under high temperature and drought.

The mean ISVAD of grain area across the genotypes decreased under D and L in the first two crop seasons (Table 1), indicating that grains were more uniformly filled under D and L than the control. An increase in grain length, width and depth is associated with a decrease in grains spike^−1^^71^. Grain size increases to compensate for the reduction in yield due to reduced grains spike^−1^ for some varieties under terminal drought^73,74^. The mean grains spike^−1^, grain yield spike^−1^ and SGW decreased under D and L, and the grain length/width ratio increased compared to C, suggesting that grain width reduction contributed to grain weight reduction (Table 1). This is in agreement with a recent study that reported a 20% average reduction in grain area mainly due to a decrease in the grain width after heat shock treatment^26^. The grain length is set during early phase of grain filling (from anthesis to 10–15 days after anthesis (DAA) by mesocarp cell elongation^75,76^. During 2020–2021, plants experienced severe heat stress, therefore mean ISVAD of grain area increased under L (Fig. 7) despite a decrease in grains spike^−1^ due to poor floret and pollen fertility under high temperature^77^ and less grain setting shortly before or at anthesis^78 ^ (Figs. 7, S5), along with decreased grain yield spike^−1^ (Fig. S6). For some genotypes, high temperature at grain filling accelerates the grain filling rate upto 30 °C, presumably reflecting increased enzyme activity and metabolic processes^79^; however, it also causes truncation of the grain filling period, which may not be fully compensated by increased rate, depending on the genotype; therefore the overall effect is a reduction of grain size^18^. The spike length and spike yield components and their responses to terminal drought and high temperature were taken into account (Fig. 3) to identify similar genotypes for assessing the contrast in only ISVAD of grain size parameters.

The posthoc test shows that among the genotypes Sunstar and Raj 4079, which are similar in spike yield components (Fig. 3), the mean ISVAD of grain area of Sunstar was significantly lower than Raj 4079 under L environment of three crop seasons (Tables S2–S4; Fig. 7). The C environments of 2018–2019 and 2019–2020 were cooler and more favorable than the other 7 environments (D and L of 2018–2019 and 2019–2020; and C, D and L of 2020–2021). As Sunstar normally has smaller grains than Raj 4079 (Fig. S10), the mean SGW of Raj 4079 was significantly higher than Sunstar under the C of 2018–2019 and 2019–2020. The SGW of Raj 4079 and Sunstar became comparable under the less favorable 7 environments which suggest that lower ISVAD of grain area of Sunstar under stress could be associated with the more stable SGW (HSI = 0.35) in contrast to SGW of Raj 4079 (HSI = 1.75) which notably decreased under L (Tables S2–S4). However, further screening of large set of germplasm is required to confirm the hypothesis that genetic variability in ISVAD of grain area could be one of the opportunities to improve the grain weight spike^−1^.

Barrero et al. reported that grain size stability is less impacted by heat stress in the genotypes with smaller grains^26^. In the present study, the genotypes which have the smaller grain size Chinese spring (15.67 mm^2^), WH 730 (17.89 mm^2^) and PBW 396 (19.49 mm^2^) have a stable ISVAD of grain area and genotypes with larger grains such as PBW 175 (23.04 mm^2^), and HD 2987 (21.62 mm^2^) are highly unstable (Figs. S10, 4). The correlation (R^2^) between grain width and grain length across all the environments in three years was 0.45 (Fig. S11). Similarly, an earlier study reported that the grain width, length, and area quantified in 210 European wheat accessions showed strong positive correlations^80^. However, a low correlation (0.29) was found under non-stressed conditions by Philipp et al.^6^. Theoretical models suggest that improving grain size and shape could boost milling yield, with large and spherical grains being the optimum grain morphology^81^. Grain *circularity* across all the genotypes and environments in three crop seasons ranged from 0.15 to 0.92, and *roundness* ranged from 0.22 to 0.89. The mean circularity and roundness of grains under the C environment of three years were 0.72 ± 0.0009 and 0.48 ± 0.0006, respectively. Under D, grains' mean circularity (0.69 ± 0.0011) and roundness (0.44 ± 0.0008) decreased by 4.2% and 8.3%, respectively. Also, under L, grains' mean circularity (0.71 ± 0.0009) and roundness (0.45 ± 0.0007) decreased by 1.4% and 6.3%, respectively. The variation in grain size and shape has been supported by the genetic and phenotypic structure, where grain size has progressively increased through changes both in grain width and length, and later modifications in grain shape have primarily been made through changes to grain length^54^.

The photosynthate availability (source capacity) is adequate for grain development, and assimilating (sink) capacity determines the grain yield^9,10,82^. Under heat and drought stress, grain filling is supported by stem reserve mobilization contributing up to 70% of grain weight to the grains, compensating for reduced leaf photosynthesis depending on the genotype^19,83^. However, in a recent study, half spike removal increased, but defoliation decreased single grain weight, indicating that grain yield is co-limited by source and sink capacity^82^. Nonetheless, there is evidence for spikelet-to-spikelet competition for assimilates within a spike leading to the unbalanced distribution of grains per spikelet along the length of the spike^80,85^. Few studies have stated that the limiting factor for reaching maximum grain weight in distal spikelets could be due to the impediments in loading of photosynthates into grains and restrictions in the transport of assimilate to grains within spike and spikelets^86–88^. Barrero et al. reported that heat stress reduces grain size by affecting assimilate translocation and grain growth rate^26^. An earlier study also stated that the source is non-limiting under mild heat stress (30/25 °C), and the grain set and single grain weight reduction is probably due to the inhibition of H^+^-ATPase activity and active transport of hexose into the endosperm^89^. Rachis plays an important role in the translocation of assimilates within the spike. Rachis parameters were determined to find out the effect of rachis size on the intra-spike assimilate distribution to the grains. Therefore, the genetic variation in the rachis parameters could be one of the factors affecting the ISVAD of grain parameters. The K means cluster plot of rachis area and perimeter of the spikes in the present study grouped the low ISVAD of grain size genotypes (Sunstar, Chinese Spring, Halna, HD 2987 and HD 2643) in the same cluster (Fig. 5). Although the assimilate supply within the spike is governed by competition among the different parts of the spike^90^, the genetic advancement in yield gain has no direct association with the vasculature size of the spike^91^. In summary, the present study could differentiate sensitive and tolerant genotypes for their intra-spike variation in grain traits under high temperature and low soil moisture conditions relative to plants grown under optimum temperature and sufficient soil moisture. The reduction in grain width contributed to the reduction in grain area, SGW, grain circularity and roundness under stress. The genetic variation in the rachis size parameters could be one of the factors affecting the ISVAD of grain size parameters.

### Genes associated with responses of distinct genotypes

RNA sequencing was carried out to identify the genes involved in uniform intra-spike assimilate distribution by comparing gene expression profiles of genotypes contrasting in ISVAD of grain area. Grain growth and development is divided into three phases^92,93^ lag-phase (from anthesis to about 10–15 DAA); filling-phase (from 15 to 35–40 DAA); and maturation phase. During the lag-phase, endosperm cells undergo cell division and cell differentiation^75,76^. The filling-phase consists of the accumulation of assimilates into the endosperm (mainly starch and proteins) and dry mass content increases. During filling-phase, cell layers of different maternal tissues exhibit degeneration processes at discrete time points^94^. The outer tissues such as vascular bundles and nucellar epidermis remain alive for the duration of starch accumulation as they play a role in nutrient transport and grain filling. Retaining live cell layers of these tissues at late stages of grain filling affect the final grain weight. Therefore, mid of filling-phase was selected for RNA sequencing of spike to identify the differentially expressed genes in genotypes contrasting in ISVAD of grain area.

As the ISVAD of grain area of Sunstar was significantly lower than Raj 4079 under L environment, the up and downregulated genes and isoforms at p < 0.05 are discussed for L only. The transcripts of three Bidirectional sugar transporter SWEET were downregulated in Raj 4079 as compared to Sunstar. The transcripts of three other proteins that are integral component of membrane involved in carbohydrate transport were downregulated in Raj 4079 as compared to Sunstar. These genes could be involved in a more efficient intra-spike assimilate distribution in Sunstar. Grain size controlling factor GS5 positively regulates grain size and weight by regulating grain width, and filling in rice^95,96^. Under L, GS5 transcripts decreased in Raj 4079 (log2 fold change − 2 and − 3.5) compared to Sunstar. Grain incomplete filling 1 which encodes a cell wall invertase that regulates the hydrolysis of sucrose and has an essential role in sucrose unloading, was also downregulated in Raj 4079 (log2 fold change − 2.6 and − 2.8). At 37 °C, the mRNA levels of the enzymes in the starch synthesis pathway are considerably reduced^97^. Sucrose phosphate synthase was highly expressed in Sunstar (Fig. 8). The transcript levels of sucrose synthase were also lower in Raj 4079 as compared to Sunstar (log2 fold − 0.7 and − 1.9). The mean SGW of Sunstar is more tolerant to high temperature (HSI = 0.35) than mean SGW of Raj 4079 (HSI = 1.75) across the three years. These could be some of the reasons for significant decrease of SGW and higher ISVAD of grain area in Raj 4079 as compared to Sunstar. Hutsch et al. postulated that the adverse effect of heat stress on the grain set and SGW reduction is probably due to the inhibition of H^+^-ATPase activity and the active transport of hexose into the endosperm^89^. The H^+^-ATPase establishes a pH gradient across the plasma membrane of the sink cell that energizes the carrier-mediated active transport of hexoses from the apoplast into developing grain by H^+^-cotransport^98,99^. However, in the present study, plasma membrane ATPase involved in proton-exporting ATPase activity across the plasma membrane is upregulated in Raj 4079 as compared to Sunstar. A total of 25 genes responsive to heat, water and oxidative stress were downregulated in Raj 4079. These results indicated that the above genes play essential roles in intra-spike assimilate distribution. In summary, the involvement of the sucrose and starch metabolism genes was a possible reason for distinct responses of genotypes differing in ISVAD. For candidates with a significant role in crop response to ISVAD of grain size under high temperature/drought, genome editing techniques can be applied to overexpress the gene and/or introduce beneficial alleles into an elite, high yielding cultivar. Understanding the genetic basis of diversity in wheat grain size and shape is critical to increasing yield potential and processing performance. Improvement of wheat grain yield should consider insight into the mechanisms of grain development and the supply of assimilates for developing grains.

### Proposed phenomics protocol for ISVAD

**Figure 9:**
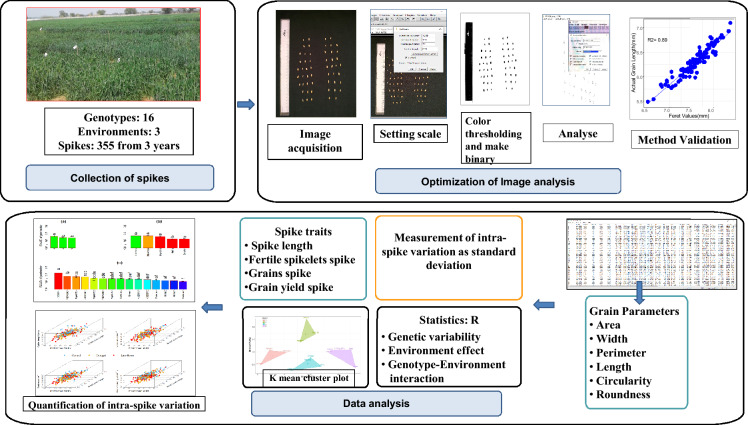
Pictorial illustration of the process of phenotyping.

We demonstrated an affordable plant phenomics approach for ISVAD using images acquired by smartphone and open-source software for image analysis. The results proved that it is possible to quantify intra-spike genetic variation in wheat grain size by employing an image-based phenomics approach.This method can be useful in routine selection as well as phenotyping for identification of genes and QTLs associated with intraspike assimilation and hence productivity of wheat under stress conditions (Fig. 9).

## Materials and methods

### Plant material and growth conditions

A set of 16 diverse wheat genotypes were seeded at the Farm Science Center, Banasthali Vidyapith, India situated at an elevation of 1010 ft. at 26°24′N, 75° 47′E (Table 2). We obtained the seeds of AKAW 4627, Chinese spring, Halna, HD 2643, HD 2967, HD 2987, HUW 234, MP 4010, PBW 175, PBW 396 and WH 730 from ICAR-Indian Institute of Wheat and Barley Research. The Department of Plant Breeding and Genetics, Punjab Agricultural University provided Sunstar. HPBW 01, Raj 3765, Raj 4037 and Raj 4079 were provided by Farm Science Centre, Banasthali Vidyapith, Tonk. The genotypes vary in spike morphology and are released for cultivation either for irrigated and timely sown environments (Raj 4037), restricted soil moisture environments (HD 2967, HD 2987, PBW 175 and PBW 396) and for delayed sowing (Halna, HD 2643, HPBW 01, HUW 234, MP 4010, Raj 3765, WH 730, AKAW 4627, Raj 4079 and Sunstar). The different spike shapes have different orientation of grains along the rachis of the spike which affects the assimilate distribution. Spike compactness has been reported to be closely related to grains spike^−1^ and thousand grain weight^100,101^.

**Table 2:** Pedigree, origin, year of release and spike shapes of wheat genotypes used in the experiment.

S.No	Genotype	Origin (year of release)	Spike shape
1	AKAW 4627	PDKV, Akola (2011)	Compact
2	Chinese Spring	China (landrace)	Compact
3	HALNA	CSUAT, Kanpur (2001)	Normal
4	HD 2643	IARI, New Delhi (2002)	Spelt
5	HD 2967	IARI, New Delhi (2010)	Spelt
6	HD 2987	IARI, New Delhi (2012)	Normal
7	HPBW01	PAU, Ludhiana (2015)	Normal
8	HUW 234	BHU, Varanasi (1984)	Compact
9	MP 4010	JNKVV Jabalpur (2003)	Spelt
10	PBW 175	PAU, Ludhiana (1989)	Normal
11	PBW 396	PAU, Ludhiana (1998)	Spelt
12	Raj 3765	Durgapura, Jaipur (1995)	Normal
13	Raj 4037	Durgapura, Jaipur (2004)	Normal
14	Raj 4079	Durgapura, Jaipur (2014)	Normal
15	Sunstar	USYD, Australia (1983)	Compact
16	WH 730	HAU, Hisar (2001)	Normal

PDKV: Dr. Punjabrao Deshmkh Krishi Vidyapeeth; CSUAT: Chandra Shekhar Azad University of Agriculture and Technology; IARI: Indian Agricultural Research Institute; PAU: Punjab Agricultural University; BHU: Banaras Hindu University; JNKVV: Jawaharlal Nehru Krishi Vishwavidyalaya; USYD: The University of Sydney; HAU: Hisar Agriculture University.

The experiment was laid out in a split-plot arrangement and randomized complete block design in 2018–2019, 2019–2020 and 2020–2021 growing seasons. The genotypes were sown within the optimum sowing period, also known as timely sown in India (generally referred to as free from heat stress) from mid of November to the last week of November, for the non-stress or control (C) and terminal drought environment (D)^34^. The sowing was done on 23 November for C and D. The late flowering genotypes under C and D were sown 8–9 days before the early flowering genotypes to synchronize anthesis during drought. Under the timely sown C and D environments, the average temperature during the vegetative growth phase was same for the late flowering and early flowering genotypes. Synchronization of anthesis helped with ease of data collection and exposure of high temperature and drought treatment was same post-anthesis for all the genotypes. The sowing between the mid of December to last week is known as late sown (L) in the Indian context when the crop is naturally exposed to terminal high-temperature stress^34^. The sowing under the L environment was carried out on 15 December. Based on local practices^102^, C and L were given six irrigations through flooding provided at 20–25, 45–50, 65–66, 80–81, 90–95 and 110–115 days after sowing (DAS). Irrigation in D was given at 20–25, 45–50 and 65–66 DAS. In the K-Means cluster plot of spike length and spike yield components, Sunstar and Raj 4079 are in same cluster but significantly differ in ISVAD of grain area under C and L environments (Tables S3, S4; Fig. 3). Halna has the lowest ISVAD of grain area under D environment which is significantly lower than same cluster genotype Raj 4079 (Table S2). PBW 396 has a consistently significantly higher ISVAD of grain area than Sunstar across the 6 environments (Tables S4–S6). HD 2987 was selected for further experiments due to the highest ISVAD under C, and also a highly unstable ISVAD across the 6 environments (Fig. 4). The remaining 11 genotypes were not significantly varying in ISVAD of grain area across the 6 environments. The ISVAD of grain area values of remaining 11 genotypes are in between the extreme values of selected 5 genotypes. Therefore, five genotypes (Sunstar, Halna, Raj 4079, HD 2987 and PBW396) were selected for third year experiments in 2020–2021. Each experimental genotype was sown in triplicate on a 2 m^2^ plot; each plot had four rows with row spacing of 22.5 cm, a row length of 200 cm, and the seeding rate was 100 kg ha^−1^. The pH, electrical conductivity, and organic carbon of the top 15 cm of soil were 7.8, 0.76 sm^−1^, and 0.47%, respectively. The soil's N, P, and K contents were 224.3, 33.06, and 362.88 kg ha^−1^, respectively. The soil's micronutrients Fe, Mn, Cu, and Zn contents were 4.03, 3.60, 1.31, and 2.31 ppm, respectively. Recommended quantities of N (60 kg ha^−1^ pre-sowing and 60 kg ha^−1^ at 40 DAS) and P (60 kg ha^−1^ pre-sowing) were applied. Manual weeding was performed at 15 and 35 days after sowing to keep weeds below the economic threshold level.

### Environment monitoring and plant measurements

The daily temperature in the field was recorded at 8:30 a.m. and 5:30 p.m. The ambient temperatures were obtained from the weather measuring equipment (Davis Vantage PRO2) installed at the adjacent field by the National Collateral Management Services Limited, Crop and Weather Intelligence Group (Table 3). The soil moisture was measured at heading stage when the irrigation was stopped in drought experiment to confirm that plants experience drought stress as compared to control. At the heading stage, the soil moisture was calculated by the gravimetric method. The soil moisture at heading stage in C, D and L was 18% (w/w), 4% (w/w) and 19% (w/w) averaged for 2018–2019 and 2019–2020. In 2020–2021, the soil moisture in C, D and L was 18% (w/w), 5% (w/w) and 16% (w/w).

**Table 3:** Monthly temperatures (°C) across the growing period in 2018–2019, 2019–2020 and 2020–2021.

Year	Month	Mean minimum temperature	Mean maximum temperature	Minimum temperature	Maximum temperature	Days > 35 °C
2018–2019	December	6.66	22.34	1.8	28	0
	January	6.98	25.48	4.1	29.5	0
	February	9.49	25.63	4.3	32.4	0
	March	14.26	31.43	9.2	41.1	6
2019–2020	December	7.33	21.18	1.6	26	0
	January	7.89	21.59	3.5	26.1	0
	February	9.44	26.45	5.8	31.8	0
	March	14.88	31.31	10.2	40.1	5
2020–2021	December	8.86	25.01	3.3	29.8	0
	January	7.64	25.48	4.1	29.5	0
	February	11.97	28.71	7.4	34.9	0
	March	15.88	35.80	11.8	41.6	23

At physiological maturity, when the spikes lost the green colour, three main shoot spikes were sampled from each plot. The number of spikelets spike^−1^, grains spike^−1^, and grain weight spike^−1^ (g) were determined. Spike length (cm) was measured, excluding awns. Grain yield plot^−1^ was measured in 2018–2019. Heads were visually classified into average, spelt and compact shapes^103^.

### Image acquisition

At physiological maturity, three main shoot spikes of each genotype and environment were collected. Each grain of a spike was manually separated from the rachis and arranged corresponding to the spikelet position with the ventral grain surface downward and no contact between grains. Images were acquired with a Redmi Note 6 Pro smartphone with a fixed distance of 31 cm between the camera and the grains placed on a dark background with a scale at the side (Fig. S12). The total data set of three crop seasons consisted of 333 images (144 images for each of first two years, and 45 images in third year) of over 18,000 grains. Rachis images were also captured separately in the same way (Fig. S13).

### Image analysis

Open-source software ImageJ version 1.46r was used to extract the features of grains from the images. A set of images (in JPEG format) were imported for processing simultaneously using the *image sequence* tool (*File* > *Import* > *Image Sequence*). The number of images processed in one session is limited as the available memory of ImageJ is 640 MB. A known length of a line was drawn along the scale in the first image. Using *set scale* tool (*Analyze* > *Set Scale*), the pixels were converted into millimeters by specifying the known length of the line in mm and clicking on checkbox “*global”* to apply this calibration to all the images. The RGB images of grains were subjected to thresholding and then converted into binary (*Process* > *Binary* > *Make Binary*) after clicking the checkbox *calculate threshold of each image*. The parameters of grains to be measured were selected using *analyze* > *Set Measurements*. The different parameters of each grain were measured using analyze particles tool (*Analyze*<*Analyze Particles*), by setting a lower limit of 2.5 mm^2^ to exclude any non-grain material. Each grain is numbered in sequence from the bottom left to the top right in an image, and an image containing numbered outlines of measured particles replaces the original image after the analysis. The grain parameters measured were *area*, *perimeter*, minimum (*MinFeret*) and maximum (*Feret*) caliper diameter, the major and minor axes of the best fitted ellipses to the grains (*Major* & *Minor)*, *Circularity* (a value between 0 to 1 for an infinitely elongated shape to a perfect circle), etc. The rachis images were also analysed in same way. The results file were saved as CSV (comma-separated values) files for analysis.

### Validation of grain length and width

The actual length and width of 100 reference grains was measured by using digital vernier calliper and these observed values were correlated with the values generated by image analysis (Figs. 1, 2). The *Feret* and *MinFeret* values from ImageJ were used as the length and width of grains. The experiment for correlation of actual grain length with *Feret* and actual grain width *MinFeret* was carried out twice to determine the consistency over estimation by ImageJ. Using the cv.lm function of the "DAAG" package of R, the predicted values were cross-validated tenfold^33^ (Figs. 1b, 2b). A k-fold cross-validation is performed for a linear model (also known as an "lm" model) via the function cv.lm. For each fold, prediction errors are computed for the observations (the "test set") and a "lm" model is fitted to all data that are not in the fold (the "training set"). The absolute error and its square make up the prediction errors. The square root of the average of the squared errors is calculated from the average prediction errors over the observations in the fold.

### Measurement of intra-spike variation as standard deviation (ISVAD)

The *MinFeret*, *Feret*, *area* (projected surface area) and *perimeter* of each grain at different locations within the spike were used for calculating the ISVAD of grain width, ISVAD of grain length, ISVAD of grain area and ISVAD of grain perimeter within a spike and within top, middle and bottom regions of spike. ISVAD was derived from variance in statistics. The standard deviation was calculated using the following formula.

$$\sigma \, = \surd (\sum \, ({\text{x}}_{{{\text{i }} }} - \upmu )^{{2}} )/\surd {\text{N}}$$

σ = Standard deviation in the grain size parameter within a spike (referred as ISVAD), N = Number of grains within a spike, x_i_ = Value of the grain size parameter of each grain within a spike, µ = Mean value of all grains within a spike.

### RNA sequencing

#### RNA extraction and high-throughput sequencing

The RNA sequencing of spike was done at 25 DAA to identify the genes involved in supply of assimilates to the developing grain and increase in dry mass content. Three biological replicates of the spike of the main stem of Sunstar and Raj 4079 were collected at 25 DAA stage under C, D and L environments in 2020–2021 and immediately snapped frozen in liquid nitrogen. Total RNA extraction was carried out using standard trizol protocol. The quality and quantity of RNA were confirmed by RNAse-free agarose gel electrophoresis, and concentration was measured using qubit, nanodrop and Agilent tapestation. High-quality RNA from all the samples was used for mRNA purification. The mRNA library was prepared using the TruSeq Stranded mRNA Kit_Plant. The libraries were then sequenced on Illumina Hiseq. X 10 platform at SciGenom Next-Gen sequencing facility, Cochin, India.

#### Preprocessing of RNA-Seq data and transcriptome profile analysis

In the preprocessing step of the raw reads, the adaptor sequences and low-quality bases were trimmed using AdapterRemoval-v2 (version 2.3.1). From the preprocessed reads, ribosomal RNA sequences were removed by aligning the reads with the silva database using bowtie2 (version 2.2.9) and subsequent workflow using samtools (version 1.9), sambamba (version 0.7.0), BamUtil (version 1.0.14) tools and in-house scripts. The preprocessed and rRNA removed reads were used for reference-based pair-wise alignment with Wheat Ensembl release 31 reference genome downloaded from Ensemble (http://ftp.ebi.ac.uk/ensemblgenomes/pub/plants/release-31/fasta/triticum_aestivum/dna/Triticum_aestivum.IWGSC1%2Bpopseq.dna.toplevel.fa.gz). The alignment was performed using the STAR program (version 2.7.6a).

#### Differential gene expression

After aligning the reads with the transcriptome, differential expression analysis for all pairs of samples comparisons was performed using the cuffdiff program of the cufflinks package (version 2.2.1). Absolute (Log2 fold change) _ 2 along with p-value < 0.05 separately were used as cut offs for identifying up and down regulated genes.

### Statistical significance

The statistical significance of the data was calculated using “agricolae” package in R version 4.0.1^104^. The Duncan’s multiple range test (DMRT) was carried out at p < 0.05 and used as a posthoc test to separate the means where ANOVA indicated significant differences.

### Statement

We confirm that experimental research and field studies on plants (either cultivated or wild), including the collection of plant material, comply with relevant institutional, national, and international guidelines and legislation.

### Supplementary Information

Supplementary Information.

## Data Availability

The datasets presented in this study can be found in online repositories and Supplementary material. The transcriptome data can be found in the National Genomics Data Centre (NGDC) database BioProject: PRJNA918542.
