# Supplementary material for: Image facilitated assessment of intra-spike variation in grain size in wheat under high temperature and drought stress
Source: Sci Rep. 2023 Nov 13;13:19850. doi: 10.1038/s41598-023-44503-x (PMC10645968; doi:10.1038/s41598-023-44503-x)
Supplement: Supplementary file 1 — Supplementary Information. [file 41598_2023_44503_MOESM1_ESM.doc]

**Supplementary Files**

**Table S1.** Analysis of variance of the intra-spike variation as standard deviation (ISVAD) of grain width, length, area and perimeter under control, terminal drought and late sown environments in 2018-2019 and 2019-2020.

| Variation | Df | ISVAD of width | ISVAD of length | ISVAD of area | ISVAD of perimeter |
| --- | --- | --- | --- | --- | --- |
| Year | 1 | 0.293*** | 0.004 | 4.175*** | 1.68207** |
| Environment | 2 | 0.017** | 0.017 | 9.674*** | 0.138 |
| Genotype | 15 | 0.014*** | 0.069*** | 2.611*** | 0.75215*** |
| Year:Environment | 2 | 0.01424** | 0.07248*** | 0.2025 | 0.77697* |
| Year:Genotype | 15 | 0.004* | 0.010 | 0.337 | 0.10999 |
| Environment:Genotype | 30 | 0.005* | 0.012. | 0.593*** | 0.17109 |
| Year:Environment:Genotype | 30 | 0.00457* | 0.02322*** | 0.4814** | 0.32103*** |
| Residuals | 190 | 0.003 | 0.008 | 0.233 | 0.17356 |

Where, ***, ** and * depicts significant differences at p ≤ 0, 0.001, 0.01, respectively.

**Table S2.** Genetic variation in image derived grain parameters, grains spike^-1^, yield spike^-1^ and single grain weight under terminal drought (D) during 2018-2019 and 2019-2020.

| Genotype | ISVAD of width | ISVAD of length | ISVAD of area | Grains spike^-1^ | Yield spike^-1^ (g) | SGW (g) |
| --- | --- | --- | --- | --- | --- | --- |
| AKAW4627 | 0.366 ± 0.020 bcd | 0.620 ± 0.028 abcdef | 3.154 ± 0.243 bc | 63 ± 4 cd | 2.65 ± 0.14 ab | 0.043 ± 0.002 a |
| Chinese spring | 0.349 ± 0.029 bcd | 0.506 ± 0.025 f | 2.441 ± 0.131 de | 74 ± 7 a | 2.12 ± 0.46 bc | 0.027 ± 0.004 bc |
| Halna | 0.280 ± 0.020 e | 0.502 ± 0.016 f | 2.046 ± 0.122 e | 47 ± 2 gh | 1.73 ± 0.20 cd | 0.037 ± 0.003 abc |
| HD2643 | 0.371 ± 0.023 abcd | 0.651 ± 0.080 abcd | 3.045 ± 0.234 bc | 46 ± 2 gh | 1.91 ± 0.30 c | 0.042 ± 0.006 a |
| HD2967 | 0.381 ± 0.034 abc | 0.637 ± 0.064 abcde | 3.139 ± 0.107 bc | 60 ± 2 cde | 2.88 ± 0.19 a | 0.047 ± 0.002 a |
| HD2987 | 0.330 ± 0.017 cde | 0.516 ± 0.036 ef | 2.409 ± 0.168 de | 53 ± 7 efgh | 1.49 ± 0.34 cd | 0.027 ± 0.003 bc |
| HPBW01 | 0.405 ± 0.028 ab | 0.645 ± 0.058 abcd | 3.413 ± 0.348 ab | 72 ± 5 ab | 2.81 ± 0.34 a | 0.038 ± 0.003 ab |
| HUW234 | 0.426 ± 0.015 a | 0.729 ± 0.024 a | 3.760 ± 0.363 a | 64 ± 3 bc | 3.03 ± 0.26 a | 0.047 ± 0.002 a |
| MP4010 | 0.321 ± 0.021 de | 0.604 ± 0.071 bcdef | 2.835 ± 0.101 cd | 44 ± 9 h | 2.11 ± 0.43 bc | 0.049 ± 0.007 a |
| PBW175 | 0.320 ± 0.019 de | 0.567 ± 0.046 cdef | 2.812 ± 0.192 cd | 44 ± 4 h | 2.02 ± 0.35 c | 0.044 ± 0.004 a |
| PBW396 | 0.386 ± 0.018 abc | 0.703 ± 0.043 ab | 3.507 ± 0.191 ab | 47 ± 2 gh | 1.89 ± 0.21 c | 0.040 ± 0.003 a |
| Raj3765 | 0.373 ± 0.022 abcd | 0.590 ± 0.037 bcdef | 3.083 ± 0.268 bc | 48 ± 1 fgh | 1.85 ± 0.15 c | 0.039 ± 0.003 ab |
| Raj4037 | 0.342 ± 0.029 cd | 0.533 ± 0.044 def | 2.732 ± 0.157 cd | 44 ± 8 h | 1.89 ± 0.23 c | 0.045 ± 0.004 a |
| Raj4079 | 0.345 ± 0.025 cd | 0.653 ± 0.056 abcd | 2.699 ± 0.302 cd | 50 ± 4 efgh | 1.23 ± 0.15 d | 0.025 ± 0.004 c |
| Sunstar | 0.360 ± 0.019 bcd | 0.562 ± 0.042 cdef | 2.568 ± 0.332 cde | 54 ± 4 defg | 2.00 ± 0.18 c | 0.037 ± 0.004 abc |
| WH730 | 0.315 ± 0.019 de | 0.670 ± 0.045 abc | 2.755 ± 0.201 cd | 56 ± 2 cdef | 2.04 ± 0.18 c | 0.036 ± 0.004 abc |

The letters ^(a-h)^ suffixed with the mean values of 6 replications (3 replications x 2 years) were derived from Duncan’s multiple range test (p < 0.05) and the means with different letters within each column are significantly different.

**Table S3.** Genetic variation in image derived grain parameters, grains spike^-1^, yield spike^-1^ and single grain weight under late sown (L) during 2018-2019 and 2019-2020.

| Genotype | ISVAD of width | ISVAD of length | ISVAD of area | Grains spike^-1^ | Grain yield spike^-1^ (g) | SGW (g) |
| --- | --- | --- | --- | --- | --- | --- |
| AKAW4627 | 0.387 ± 0.017 a | 0.695 ± 0.042 a | 3.395 ± 0.239 a | 72 ± 2 a | 3.19 ± 0.12 a | 0.044 ± 0.002 abcd |
| Chinese spring | 0.316 ± 0.026 ab | 0.508 ± 0.038 de | 2.198 ± 0.104 de | 66 ± 7 ab | 1.27 ± 0.17 i | 0.022 ± 0.005 f |
| Halna | 0.343 ± 0.025 ab | 0.528 ± 0.036 cde | 2.442 ± 0.161 cd | 49 ± 3 de | 2.04 ± 0.21 def | 0.042 ± 0.004 abcd |
| HD2643 | 0.311 ± 0.023 ab | 0.572 ± 0.030 bcd | 2.655 ± 0.220 bcd | 47 ± 3 e | 1.91 ± 0.02 efgh | 0.042 ± 0.003 abcd |
| HD2967 | 0.353 ± 0.043 ab | 0.576 ± 0.034 abcd | 2.678 ± 0.133 bcd | 63 ± 1 ab | 2.57 ± 0.09 bc | 0.041 ± 0.001 bcd |
| HD2987 | 0.363 ± 0.057 a | 0.600 ± 0.058 abcd | 2.671 ± 0.263 bcd | 44 ± 6 e | 1.98 ± 0.22 efg | 0.047 ± 0.003 abc |
| HPBW01 | 0.345 ± 0.016 ab | 0.649 ± 0.033 ab | 3.216 ± 0.266 ab | 61 ± 3 bc | 2.88 ± 0.11 ab | 0.048 ± 0.002 abc |
| HUW234 | 0.387 ± 0.015 a | 0.683 ± 0.072 ab | 3.299 ± 0.202 a | 72 ± 2 a | 2.86 ± 0.06 ab | 0.040 ± 0.001 bcd |
| MP4010 | 0.365 ± 0.019 a | 0.630 ± 0.044 abc | 3.074 ± 0.190 ab | 42 ± 4 e | 1.92 ± 0.20 efgh | 0.047 ± 0.004 abc |
| PBW175 | 0.336 ± 0.054 ab | 0.595 ± 0.047 abcd | 3.099 ± 0.295 ab | 46 ± 11 e | 2.10 ± 0.35 cdef | 0.051 ± 0.006 ab |
| PBW396 | 0.347 ± 0.020 ab | 0.604 ± 0.034 abcd | 2.990 ± 0.185 abc | 41 ± 3 e | 1.53 ± 0.13 ghi | 0.038 ± 0.005 cd |
| Raj3765 | 0.315 ± 0.019 ab | 0.621 ± 0.031 abcd | 2.979 ± 0.172 abc | 51 ± 3 cde | 2.37 ± 0.16 cde | 0.046 ± 0.002 abc |
| Raj4037 | 0.329 ± 0.041 ab | 0.459 ± 0.037 e | 2.325 ± 0.198 d | 42 ± 5 e | 2.15 ± 0.14 cde | 0.053 ± 0.003 a |
| Raj4079 | 0.311 ± 0.041 ab | 0.615 ± 0.018 abcd | 2.696 ± 0.148 bcd | 47 ± 4 e | 1.64 ± 0.19 fghi | 0.035 ± 0.003 de |
| Sunstar | 0.280 ± 0.024 b | 0.452 ± 0.034 e | 1.778 ± 0.092 e | 59 ± 2 bcd | 1.48 ± 0.13 hi | 0.025 ± 0.002 ef |
| WH730 | 0.345 ± 0.019 ab | 0.650 ± 0.021 ab | 3.077 ± 0.169 ab | 59 ± 4 bcd | 2.42 ± 0.16 cde | 0.040 ± 0.003 bcd |

The letters ^(a-e)^ suffixed with the mean values of 6 replications (3 replications x 2 years) were derived from Duncan’s multiple range test (p < 0.05) of  and the means with different letters within each column are significantly different.

**Table S4.** Genetic variation in image derived grain parameters, grains spike^-1^, yield spike^-1^ and single grain weight under control (C) during 2018-2019 and 2019-2020.

| Genotype | ISVAD of width | ISVAD of length | ISVAD of area | Grains spike^-1^ | Grain yield spike^-1^ (g) | SGW (g) |
| --- | --- | --- | --- | --- | --- | --- |
| AKAW4627 | 0.409 ± 0.018 abc | 0.616 ± 0.027 cde | 3.510 ± 0.184 abc | 75 ± 3 ab | 3.35 ± 0.30 ab | 0.045 ± 0.005 abc |
| Chinese spring | 0.371 ± 0.022 abcd | 0.531 ± 0.039 fg | 2.700 ± 0.303 e | 83 ± 10 a | 2.82 ± 0.21 abcd | 0.036 ± 0.004 cd |
| Halna | 0.333 ± 0.027 cd | 0.530 ± 0.043 fg | 2.700 ± 0.219 e | 58 ± 3 c | 2.47 ± 0.13 cde | 0.043 ± 0.002 abcd |
| HD2643 | 0.354 ± 0.012 abcd | 0.621 ± 0.020 cde | 3.634 ± 0.133 ab | 60 ± 1 c | 2.97 ± 0.05 abc | 0.050 ± 0.001 ab |
| HD2967 | 0.404 ± 0.029 abc | 0.642 ± 0.041 bcde | 3.484 ± 0.276 abc | 76 ± 7 ab | 3.26 ± 0.15 ab | 0.048 ± 0.002 ab |
| HD2987 | 0.420 ± 0.035 ab | 0.691 ± 0.074 bc | 4.011 ± 0.289 a | 54 ± 4 c | 2.69 ± 0.31 bcd | 0.050 ± 0.004 ab |
| HPBW01 | 0.341 ± 0.029 bcd | 0.566 ± 0.027 ef | 2.835 ± 0.142 de | 71 ± 6 b | 2.86 ± 0.15 abcd | 0.041 ± 0.002 bcd |
| HUW234 | 0.419 ± 0.031 ab | 0.780 ± 0.038 a | 3.794 ± 0.139 ab | 71 ± 5 b | 3.44 ± 0.18 a | 0.049 ± 0.002 ab |
| MP4010 | 0.401 ± 0.023 abc | 0.619 ± 0.042 cde | 3.347 ± 0.187 bcd | 48 ± 3 c | 1.98 ± 0.25 e | 0.041 ± 0.004 bcd |
| PBW175 | 0.345 ± 0.036 bcd | 0.636 ± 0.030 bcde | 3.464 ± 0.275 abc | 54 ± 4 c | 2.83 ± 0.21 abcd | 0.053 ± 0.003 a |
| PBW396 | 0.432 ± 0.035 a | 0.702 ± 0.030 b | 3.725 ± 0.149 ab | 59 ± 5 c | 2.87 ± 0.32 abcd | 0.048 ± 0.001 ab |
| Raj3765 | 0.343 ± 0.015 bcd | 0.579 ± 0.031 ef | 2.938 ± 0.189 cde | 55 ± 2 c | 2.51 ± 0.09 cde | 0.046 ± 0.002 abc |
| Raj4037 | 0.352 ± 0.011 abcd | 0.617 ± 0.044 cde | 3.244 ± 0.246 bcde | 48 ± 2 c | 2.23 ± 0.24 de | 0.046 ± 0.004 abc |
| Raj4079 | 0.329 ± 0.023 cd | 0.594 ± 0.026 def | 3.001 ± 0.147 cde | 50 ± 9 c | 2.47 ± 0.35 cde | 0.051 ± 0.003 ab |
| Sunstar | 0.304 ± 0.013 d | 0.475 ± 0.038 g | 2.142 ± 0.129 f | 74 ± 4 ab | 2.55 ± 0.16 cde | 0.038 ± 0.001 cd |
| WH730 | 0.309 ± 0.037 d | 0.668 ± 0.033 bcd | 2.833 ± 0.378 de | 59 ± 5 c | 2.24 ± 0.33 de | 0.038 ± 0.005 cd |

The letters ^(a-f)^ suffixed with the mean values of 6 replications (3 replications x 2 years) were derived from Duncan’s multiple range test (p < 0.05) of  and the means with different letters within each column are significantly different.


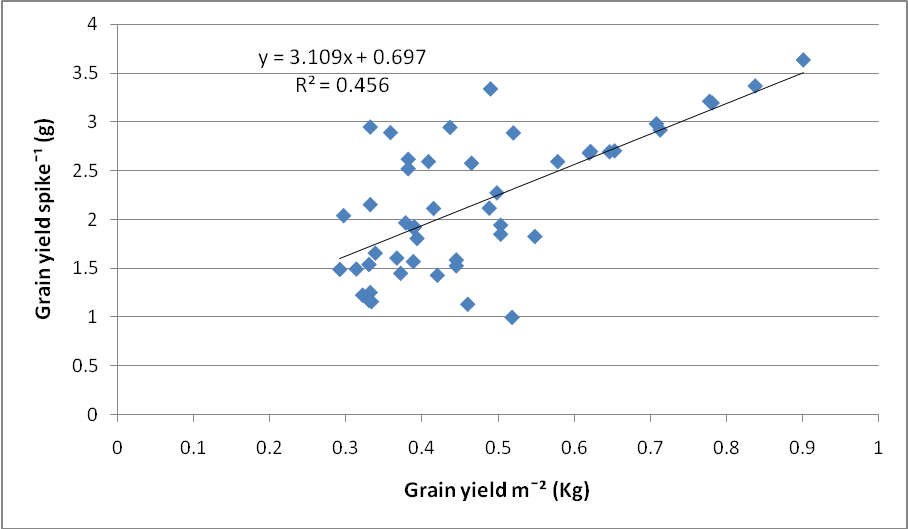


**Fig. S1.** Coefficient of determination (R^2^) between grain yield spike^-1^ and grain yield plot^-1^ of 16 genotypes under control, drought and late sown environments in 2018-2019. Each dot represents three replications of a genotype.


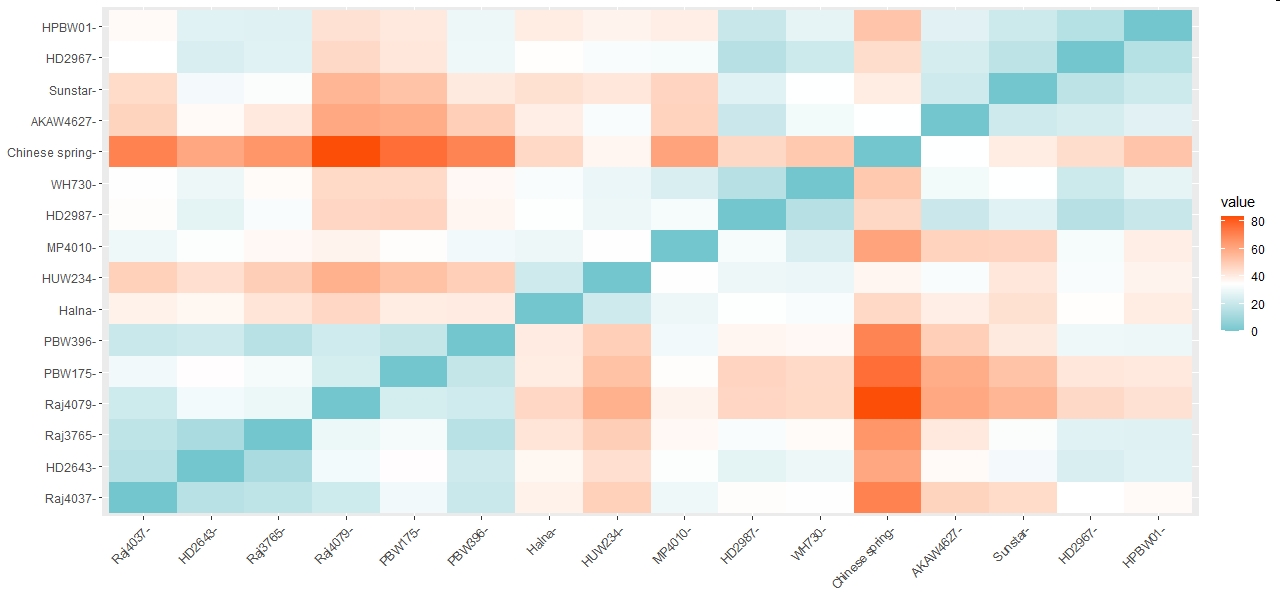


**Fig. S2.** Distance heat map of intra-spike variation as standard deviation (ISVAD) of width, length, area and perimeter of the grains within a spike under C, D and L environments (p ≤ 0.05) during 2018-2019 and 2019-2020.


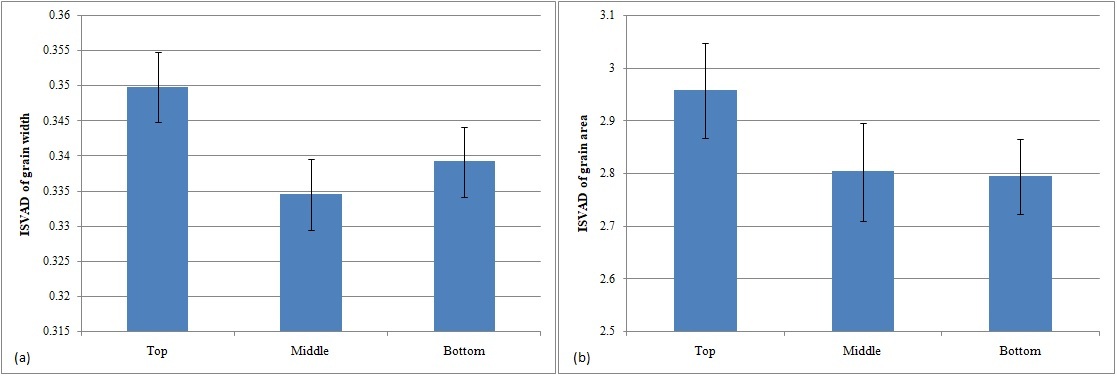


**Fig. S3.** (a) Average intra-spike variation as standard deviation (ISVAD) of grain width and (b) ISVAD of grain area in top, middle and bottom regions of spike across sixteen wheat genotypes under control environment during 2018-2019 and 2019-2020.


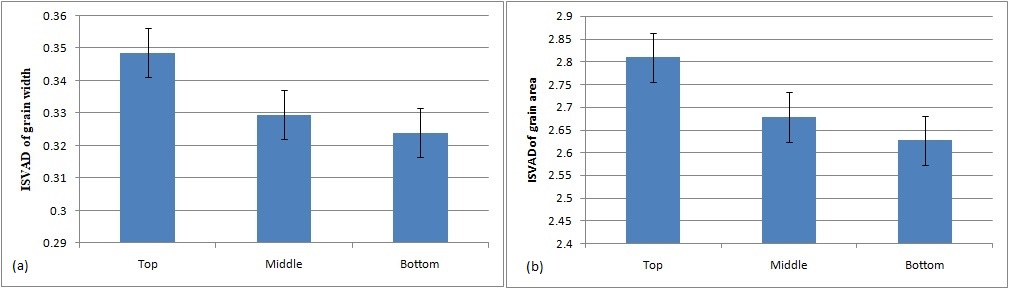


**Fig. S4.** (a) Average intra-spike variation as standard deviation (ISVAD) of grain width and (b) ISVAD of grain area in top, middle and bottom regions of spike across sixteen genotypes and six environments during 2018-2019 and 2019-2020.


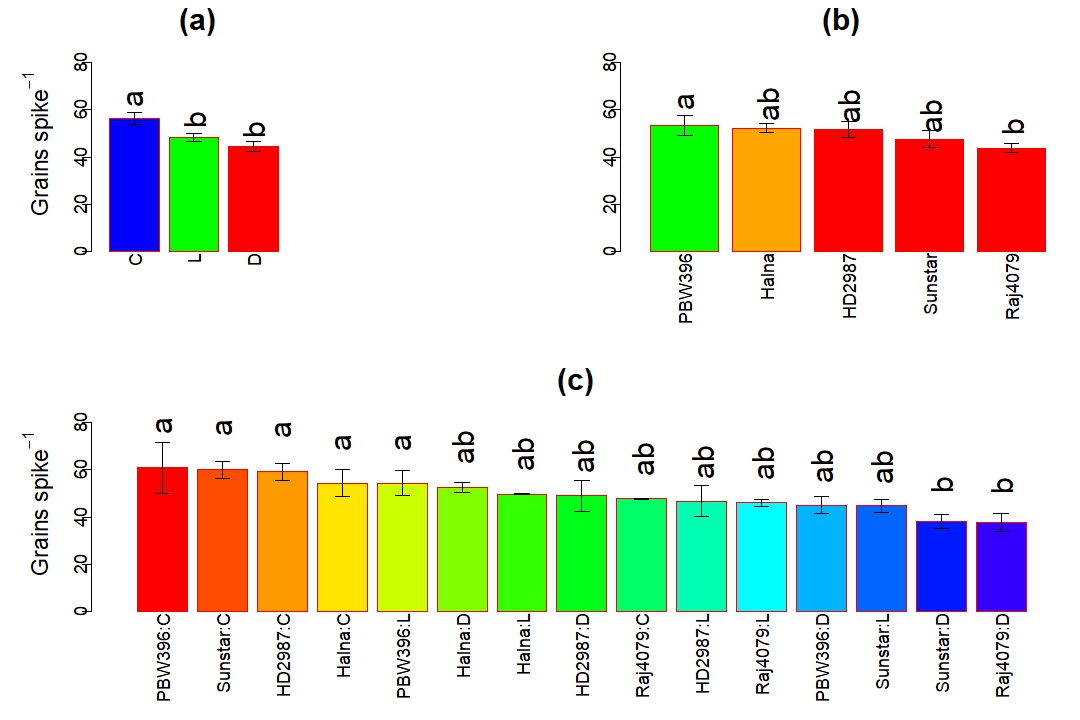


**Fig. S5.** Variation in grains spike^-1^. (a) The effect of late sown (L), terminal drought (D) and Control (C) environments on grains spike^-1^ in 2020-2021. Each bar in the environment effect represents 15 observations (3 replications and 5 genotypes). (b) The genotype effect across the environments on grains spike^-1^. Each bar in the genotype effect represents 9 observations (mean values of three replications and three environments). (c) Genotype environment interaction effect on grains spike^-1^. Each bar in the genotype environment interaction effect represents mean values of three replications. Letters represent the significance of differences among mean values as computed by the Duncan multiple range test at p ≤ 0.05. Genotypes with common letters are not significantly different.


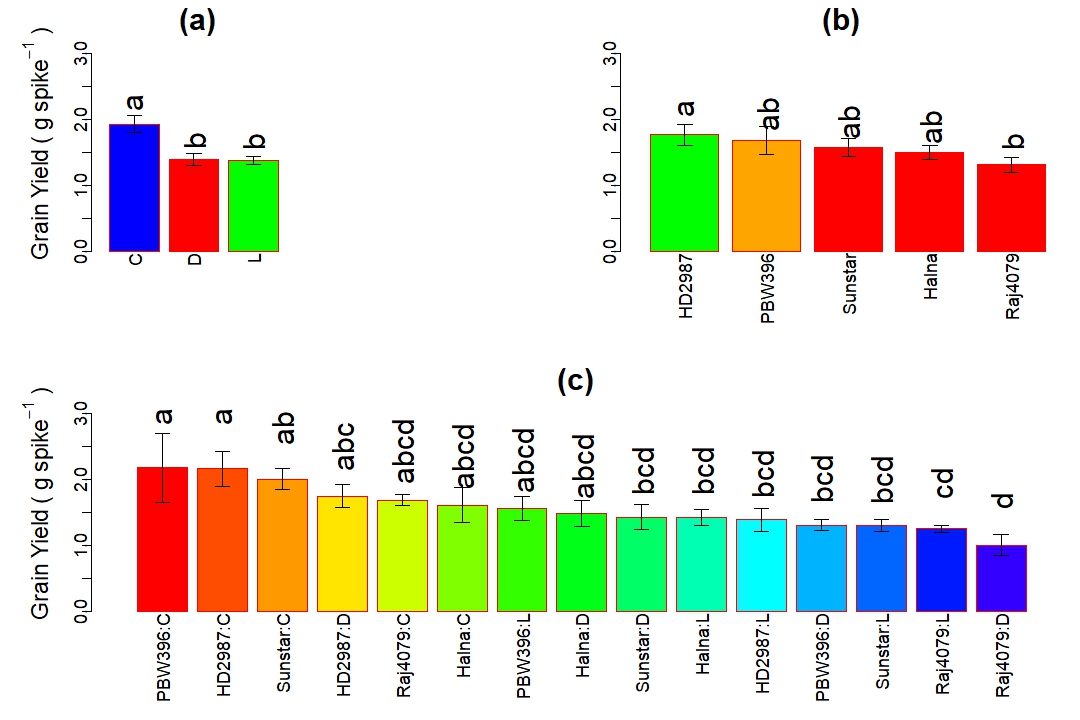


**Fig. S6.** Variation in grain yield spike^-1^. (a) The effect of late sown (L), terminal drought (D) and Control (C) environments on grain yield spike^-1^ in 2020-2021. Each bar in the environment effect represents 15 observations (3 replications and 5 genotypes). (b) The genotype effect across the environments on grain yield spike^-1^. Each bar in the genotype effect represents 9 observations (mean values of three replications and three environments). (c) Genotype environment interaction effect on grain yield spike^-1^. Each bar in the genotype environment interaction effect represents mean values of three replications. Letters represent the significance of differences among mean values as computed by the Duncan multiple range test at p ≤ 0.05. Genotypes with common letters are not significantly different.

**Table S5. Analysis of variance of intra-spike variation as standard deviation ISVAD of grain area of top, middle and bottom regions of wheat spike under control, terminal drought and late sown environments during 2018-19 and 2019-20.**

| Source of Variation | Df | Top ISVAD area | Middle ISVAD area | Bottom ISVAD area |
| --- | --- | --- | --- | --- |
| Year | 1 | 4.640** | 11.0156*** | 0.150** |
| Environment | 2 | 3.039** | 1.373. | 0.163** |
| Genotype | 15 | 2.666*** | 3.225*** | 2.196*** |
| Year:Environment | 2 | 1.187. | 0.190 | 0.008 |
| Year:Genotype | 15 | 1.022* | 0.657 | 0.458 |
| Environment:Genotype | 30 | 0.769* | 0.561 | 0.759*** |
| Year:Environment:Genotype | 30 | 0.796* | 0.847** | 0.404 |
| Residuals | 190 | 0.491 | 0.464 | 0.329 |

Where, ***, **, * and **.** depicts significant differences at p ≤ 0, 0.001, 0.01 and 0.05, respectively.

**Table S6.** Analysis of variance of intra-spike variation as standard deviation (ISVAD) of grain width of top, middle and bottom regions of wheat spike under control, terminal drought and late sown environments during 2018-2019 and 2019-2020.

| Variation | Df | Top SD width | Middle SD width | Bottom SD width |
| --- | --- | --- | --- | --- |
| Year | 1 | 0.35389*** | 0.273657*** | 0.248783*** |
| Environment | 2 | 0.01346 | 0.013514. | 0.019301* |
| Genotype | 15 | 0.0126* | 0.022014*** | 0.016139*** |
| Year:Environment | 2 | 0.04735*** | 0.001898 | 0.000409 |
| Year:Genotype | 15 | 0.01662** | 0.00881. | 0.006659 |
| Environment:Genotype | 30 | 0.00574 | 0.004683 | 0.010257** |
| Year:Environment:Genotype | 30 | 0.0081 | 0.008998 | 0.005381 |
| Residuals | 190 | 0.00654 | 0.005307* | 0.005382 |

**Table S7.** Analysis of variance of the intra-spike variation as standard deviation (ISVAD) of grain width, length, area and perimeter of five genotypes under control, terminal drought and late sown environments in 2018-2019, 2019-2020 and 2020-2021.

| Variation | Df | ISVAD of width | ISVAD of length | ISVAD of area | ISVAD of perimeter |
| --- | --- | --- | --- | --- | --- |
| Genotype | 4 | 0.0236555** | 0.308635** | 4.6087*** | 0.5915*** |
| Environment | 2 | 0.0099817 | 0.094619 | 2.0092* | 0.085 |
| Year | 1 | 0.0269152* | 0.083623 | 3.2677** | 0.11616 |
| Genotype:Environment | 8 | 0.0049149 | 0.105299 | 0.6342 | 0.15432 |
| Genotype:Year | 4 | 0.0008808 | 0.143454 | 0.1315 | 0.10993 |
| Environment:Year | 2 | 0.0073888 | 0.1609 | 2.1817** | 0.49846* |
| Genotype:Environment:Year | 8 | 0.0062029 | 0.144553. | 1.0812* | 0.27576* |
| Residuals | 105 | 0.005083 | 0.079666 | 0.4336 | 0.11551 |

Where, ***, **, * and **.** depicts significant differences at p ≤ 0, 0.001, 0.01 and 0.05, respectively.

**Table S8.** Raw fastq summary

| Sample | Read Orientation | Number of raw reads | Number of bases (Mb) | Mean Phred score | GC (%) | %data>=Q30 | Raw read length (bp) |
| --- | --- | --- | --- | --- | --- | --- | --- |
|  |  |  |  |  |  |  |  |
| RL1 | R1 | 2,83,36,184 | 4,278.76 | 35.85 | 53.12 | 93.6 | 151 |
|  | R2 | 2,83,36,184 | 4,278.76 | 35.4 | 53.18 | 91.23 | 151 |
| RL2 | R1 | 2,81,52,812 | 4,251.07 | 35.93 | 53.38 | 94.07 | 151 |
|  | R2 | 2,81,52,812 | 4,251.07 | 35.59 | 53.39 | 92.25 | 151 |
| RL3 | R1 | 2,88,18,864 | 4,351.65 | 35.96 | 55.18 | 94.22 | 151 |
|  | R2 | 2,88,18,864 | 4,351.65 | 35.77 | 55.19 | 93.16 | 151 |
| SL1 | R1 | 2,52,98,382 | 3,820.06 | 35.6 | 52.14 | 92.39 | 151 |
|  | R2 | 2,52,98,382 | 3,820.06 | 34.82 | 52.38 | 88.49 | 151 |
| SL2 | R1 | 2,84,33,397 | 4,293.44 | 35.53 | 52.05 | 92.03 | 151 |
|  | R2 | 2,84,33,397 | 4,293.44 | 34.72 | 52.29 | 87.99 | 151 |
| SL3 | R1 | 2,60,52,771 | 3,933.97 | 35.43 | 52 | 91.63 | 151 |
|  | R2 | 2,60,52,771 | 3,933.97 | 34.4 | 52.3 | 86.46 | 151 |

Where RL- Raj 4079 late sown, SL- Sunstar late sown, R1- Read1, R2- Read2


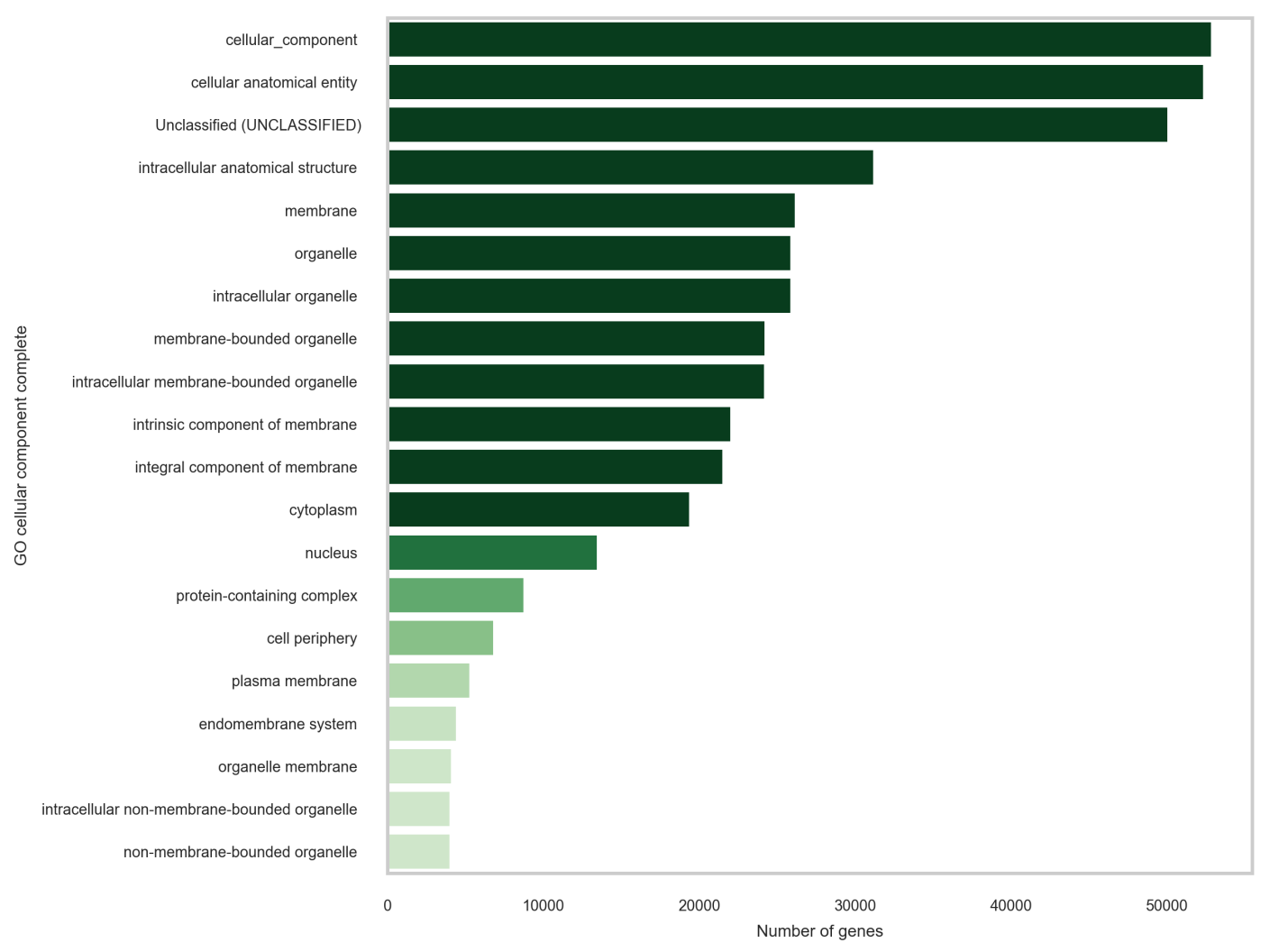


**Fig. S7.** The cellular component genes downregulated in Raj 4079 as compared to Sunstar under late sown (SL and RL).


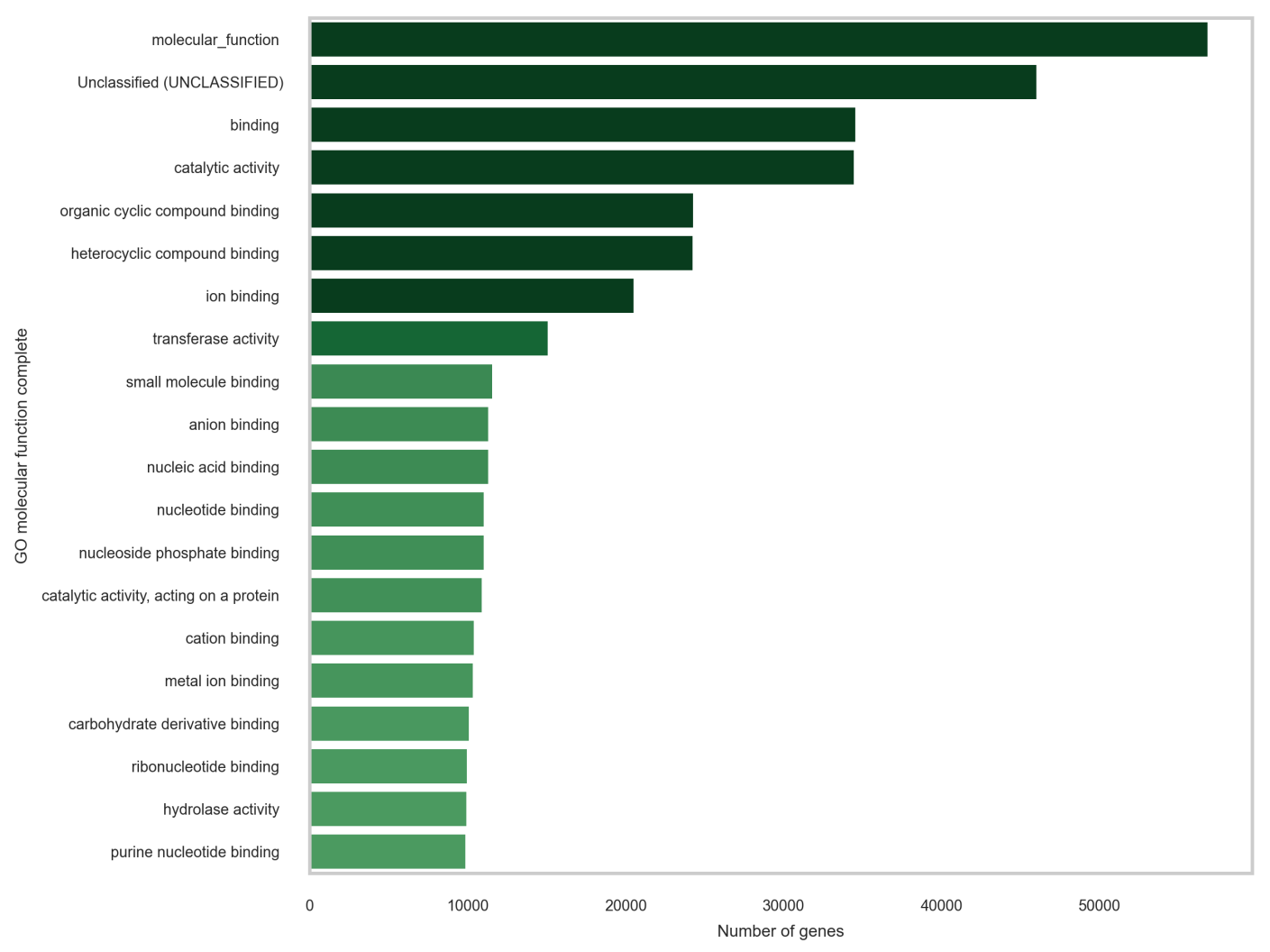


**Fig. S8.**The molecular function genes downregulated in Raj 4079 as compared to Sunstar under late sown (SL and RL).


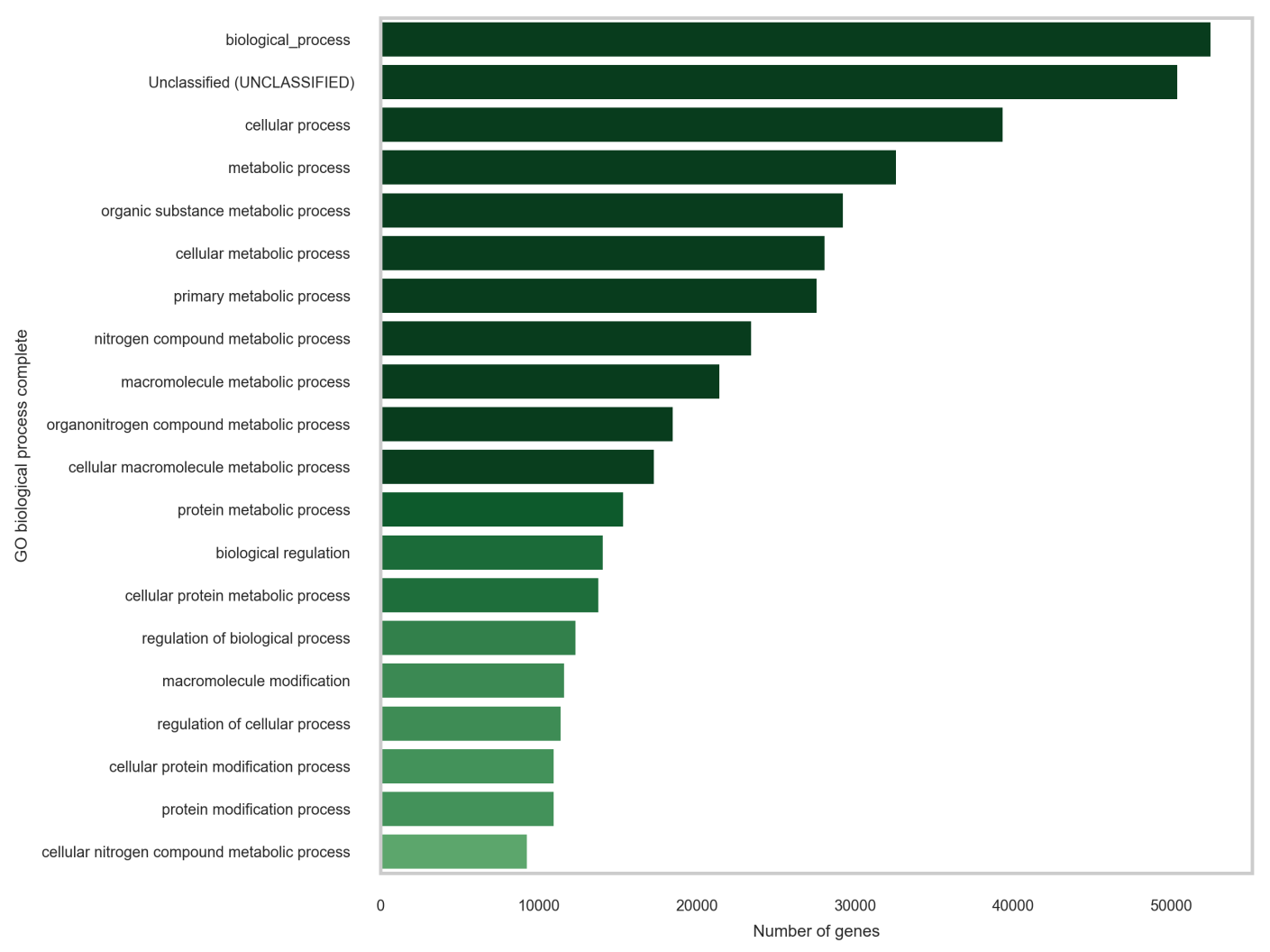


**Fig. S9.** The biological process genes downregulated in Raj 4079 as compared to Sunstar under late sown (SL and RL).


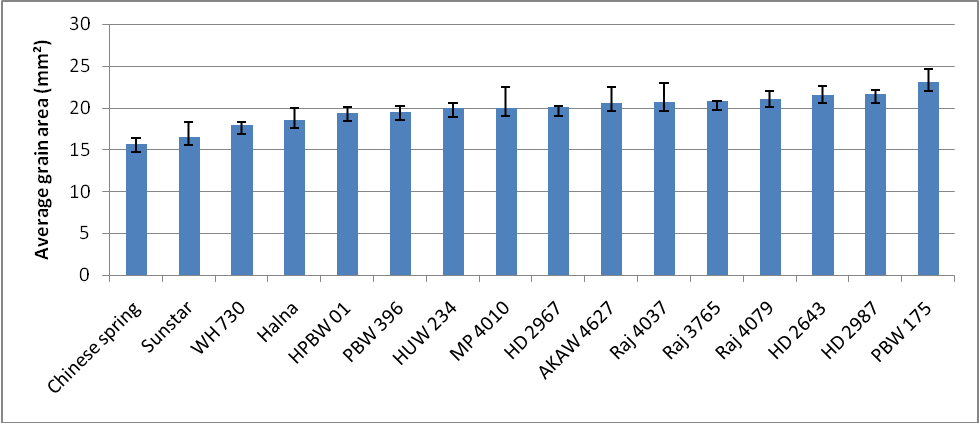


**Fig. S10.** Average grain area (mm²) of experimental genotypes under control for 2018-2019 and 2019-2020.


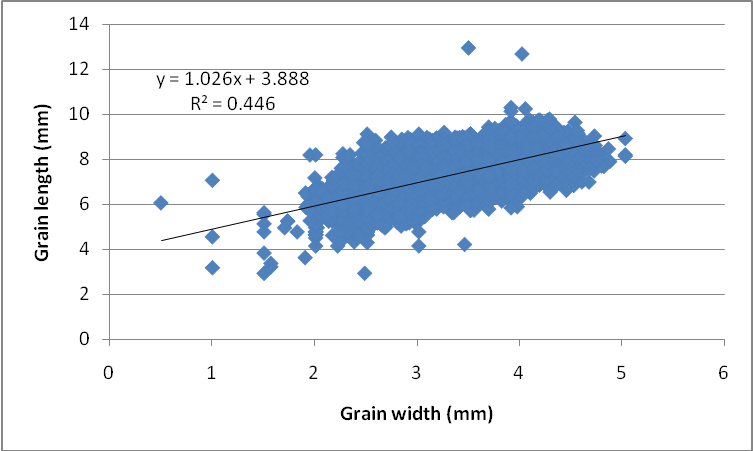


**Fig. S11.** Coefficient of determination (R^2^) between grain width and grain length of over 18000 grains of three years analysed by ImageJ.


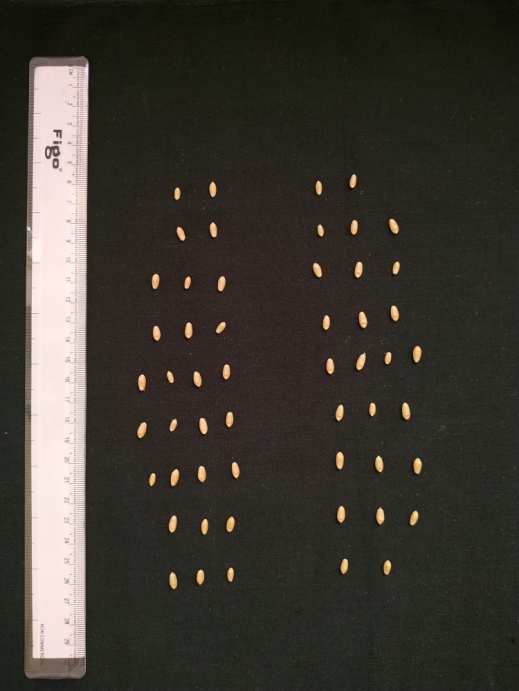


**Fig. S12.** Image acquired using redmi note 6 pro smart phone with the grains arranged according to the position in spike

**
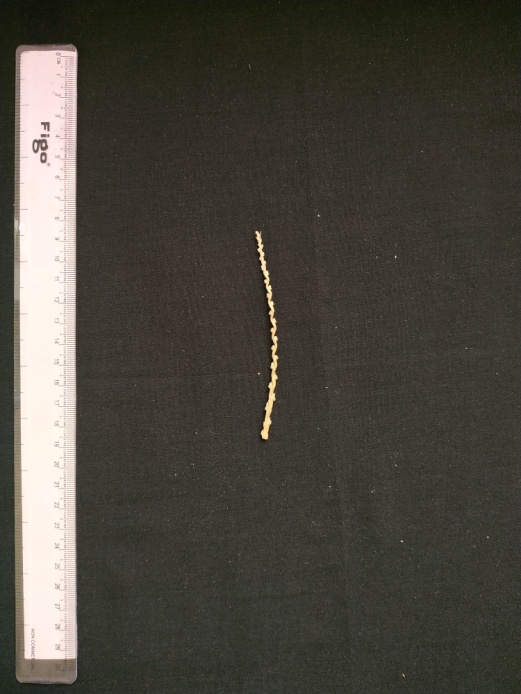
**

**Fig. S13.** Image acquired using redmi note 6 pro smart phone with the rachis of spike placed on dark background.
